# Coordination polymer glass from a protic ionic liquid: proton conductivity and mechanical properties as an electrolyte†

**DOI:** 10.1039/d0sc01737j

**Published:** 2020-04-17

**Authors:** Tomohiro Ogawa, Kazuki Takahashi, Sanjog S. Nagarkar, Koji Ohara, You-lee Hong, Yusuke Nishiyama, Satoshi Horike

**Affiliations:** AIST-Kyoto University Chemical Energy Materials Open Innovation Laboratory (ChEM-OIL), National Institute of Advanced Industrial Science and Technology (AIST) Yoshida-Honmachi, Sakyo-ku Kyoto 606-8501 Japan horike@icems.kyoto-u.ac.jp; Institute for Integrated Cell-Material Sciences, Institute for Advanced Study, Kyoto University Yoshida-Honmachi, Sakyo-ku Kyoto 606-8501 Japan; Advanced Research and Innovation Center, DENSO Corporation 500-1 Minamiyama, Komenoki-cho Nisshin Aichi 470-0111 Japan; Diffraction and Scattering Division, Center for Synchrotron Radiation Research, Japan Synchrotron Radiation Research Institute (JASRI) Kouto Sayo Hyogo 679-5198 Japan; RIKEN-JEOL Collaboration Center Yokohama Kanagawa 230-0045 Japan; JEOL RESONANCE Inc. Akishima Tokyo 196-8558 Japan; Department of Synthetic Chemistry and Biological Chemistry, Graduate School of Engineering, Kyoto University Katsura, Nishikyo-ku Kyoto 615-8510 Japan; Department of Materials Science and Engineering, School of Molecular Science and Engineering, Vidyasirimedhi Institute of Science and Technology Rayong 21210 Thailand

## Abstract

High proton conducting electrolytes with mechanical moldability are a key material for energy devices. We propose an approach for creating a coordination polymer (CP) glass from a protic ionic liquid for a solid-state anhydrous proton conductor. A protic ionic liquid (dema)(H_2_PO_4_), with components which also act as bridging ligands, was applied to construct a CP glass (dema)_0.35_[Zn(H_2_PO_4_)_2.35_(H_3_PO_4_)_0.65_]. The structural analysis revealed that large Zn–H_2_PO_4_^−^/H_3_PO_4_ coordination networks formed in the CP glass. The network formation results in enhancement of the properties of proton conductivity and viscoelasticity. High anhydrous proton conductivity (*σ* = 13.3 mS cm^−1^ at 120 °C) and a high transport number of the proton (0.94) were achieved by the coordination networks. A fuel cell with this CP glass membrane exhibits a high open-circuit voltage and power density (0.15 W cm^−2^) under dry conditions at 120 °C due to the conducting properties and mechanical properties of the CP glass.

## Abstract 2

A proton-conducting coordination polymer glass derived from a protic ionic liquid works as a moldable solid electrolyte and the anhydrous fuel cell showed *I*–*V* performance of 0.15 W cm^−2^ at 120 °C.

## Graphical abstract

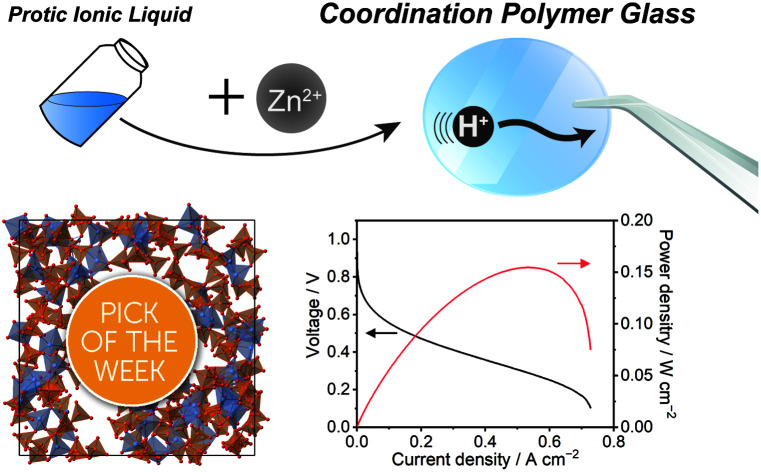

## Introduction

Proton conductive materials are important components of various electrochemical devices.^1–3^ One significant device is the fuel cell, and anhydrous proton conductors as electrolytes working at 120–200 °C have long been in high demand.^4^ In the past decade, a number of studies on the proton conductivity of coordination polymers (CPs) and metal–organic frameworks (MOFs) have been reported.^5,6^ Crystalline CP/MOFs have advantages for high proton conductivity because of their tailorable pores and ability to accommodate guest molecules. However, they are intrinsically non-moldable because of their crystalline nature, and the grain boundary causes gas leaking and additional resistance in the electrolyte layers. The preparation of mechanically stable membranes is one of the bottlenecks in electrolyte developments.^7^ More recently, CP/MOFs in liquid and glassy states have received attention as moldable amorphous materials.^8–13^ The phase transitions of CP/MOFs from crystals to liquids and glasses provide the option of use as a grain boundary-free monolith, which is a promising property for solid electrolytes.^8^ In spite of their potential, there are still a limited number of reports on proton-conductive CP/MOFs glass, and there are no reports of CP/MOFs to satisfy the criteria for sufficient proton conductivity (above 10 mS cm^−1^) under anhydrous conditions.^14^

To develop a CP glass with high intrinsic proton conductivity, we focused on protic ionic liquids which are known as representative anhydrous proton conducting liquids.^15,16^ They are a subset of ionic liquids synthesized from Brønsted acids and bases. A number of combinations of acids and bases to create protic ionic liquids have been reported, and the optimal compositions achieved high proton conductivity (reaching 50 mS cm^−1^ at 120 °C) and high chemical and thermal stabilities.^17^ Protic ionic liquids exhibiting high proton conductivity typically exhibit low viscosity.^18,19^ However, low viscosity liquids are difficult to use in membranes, which results in a lower performance.^20–23^ Furthermore, high mobility of both cations and anions results in a low transport number of protons. This low transport number leads to a decrease in the open-circuit voltage (OCV) in fuel cells.^21,24^ There is also scientific interest in the methodology to improve transport numbers while keeping the high inherent conductivity of protic ionic liquids.

**Fig. 1:**
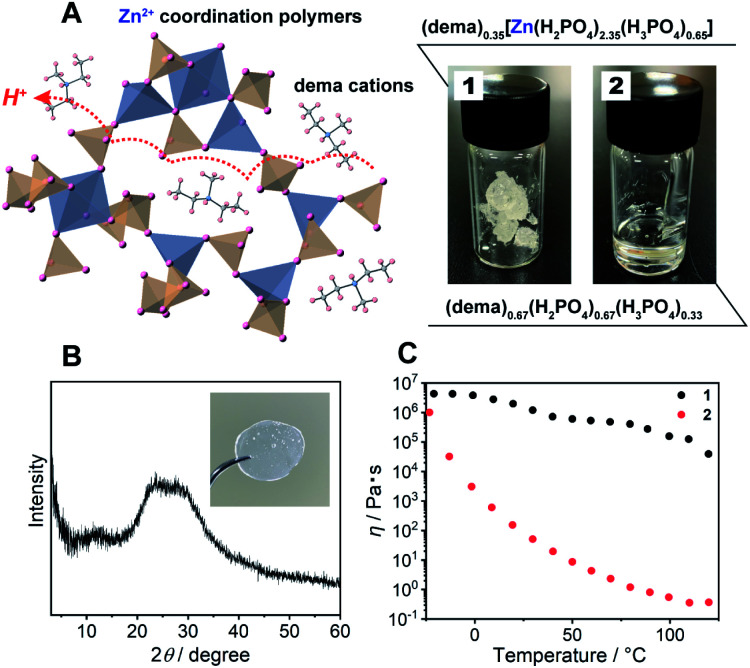
(A) Schematic structure of **1**, the blue and orange polyhedra represent Zn^2+^ and H_*n*_PO_4_, the captured organic ions indicate dema cations. The formula and digital photographs of **1** and **2** at 25 °C. (B) The powder X-ray diffraction (PXRD) pattern of **1**. The inset shows a digital photograph of a monolith of **1** after annealing at 120 °C. (C) Temperature dependence of the viscosity of **1** (black) and **2** (red).

In this study, we propose a new approach for the design of a proton conductor based on a CP glass synthesized from a protic ionic liquid and metal ions. Appropriately selected metal ions can interact with the anions to form a CP with the desirable characteristics of moldability, proton conductivity, as well as a high transport number (Fig. 1A left). To date, there have been no reports on metal ion incorporation into protic ionic liquids for the modulation of proton conducting dynamics.^16^ Here, a CP glass consisting of a protic ionic liquid (dema)(H_2_PO_4_^−^) (dema = diethylmethylammonium cation) and Zn^2+^ was synthesized as a glassy coordination polymer. The structural analysis of the coordination network in the CP glass elucidates the enhanced proton conductivity, transport number, and mechanical properties, as well as performance in a H_2_/O_2_ fuel cell under anhydrous conditions.

## Results and discussion

As a protic ionic liquid, (dema)(H_2_PO_4_) (proton conductivity of 6.5 mS cm^−1^ at 120 °C) is a candidate for further evaluation.^17^ The monoanionic H_2_PO_4_^−^ can work both as a proton carrier and a bridging ligand (Fig. 1A left). Redox inactive Zn^2+^ is used as a metal ion, which is often used for CP/MOF glasses.^8,9^ The amorphous compound containing Zn^2+^, dema, and H_2_PO_4_^−^ was synthesized by neutralization of zinc oxide (ZnO), diethylmethylamine and phosphoric acid (H_3_PO_4_) (Scheme S1† top). Grinding these materials afforded **1** as a viscoelastic solid (Fig. 1A). As a reference for **1**, we also prepared (dema)(H_2_PO_4_) (**2**) according to the method available in the literature (Scheme S1,† bottom).^17^

The chemical compositions of **1** and **2** were characterized by solid-state magic angle spinning nuclear magnetic resonance (SSNMR) (Fig. S1†) and elemental analysis. The proton peaks were assigned to either dema or H_2_PO_4_^−^/H_3_PO_4_, and both **1** and **2** contained no detectable water molecules. The observed integration value of the ^1^H signals of dema (dema : H_2_PO_4_^−^ = 1 : 7.6) was different from the theoretical chemical composition of the starting materials (dema : H_2_PO_4_^−^ = 1 : 3). Previous reports identified that highly stable protic ionic liquids have a p*K*a difference between the cation and the anion which is larger than 10.^18,25^ The difference in the p*K*a value between H_3_PO_4_ (2.1) and the diethylmethylammonium cation (10.5), Δp*K*a, was 8.4, and the neutral amine was partially evaporated during the drying process (Scheme S1†). Both SSNMR and the elemental analysis indicated an identical composition of **1**, *i.e.*, (dema)_0.35_[Zn(H_2_PO_4_)_2.35_(H_3_PO_4_)_0.65_]. Partial evaporation of the amine was also observed for **2**, even though we followed the reported synthesis method.^17^^1^H SSNMR gave the composition of **2** as (dema)_0.67_(H_2_PO_4_)_0.67_(H_3_PO_4_)_0.33_. Furthermore, the ^31^P SSNMR spectrum of **1** exhibits a single peak at 0.2 ppm (20 °C, Fig. S2†), which suggests that the phosphoric acid species remains in the form of orthophosphoric acids after drying.^26,27^ The powder X-ray (Cu Kα) diffraction (PXRD) pattern of **1** at room temperature exhibited no Bragg peaks, and **1** was assignable to an amorphous state (Fig. 1B). The IR spectrum was measured to obtain information about the proton conductive groups (Fig. S3†). However, the spectrum shape was very broad and difficult to use for assignments. This is probably due to its amorphous nature as it contains many different configurations of cations and coordination structures. The thermal properties of **1** were subsequently determined by thermogravimetric analysis (TGA) and differential scanning calorimetry (DSC, Fig. S4†). There was no weight loss up to 200 °C. In addition, the DSC curves of **1** showed a glass transition at −22 °C. It has no first-order phase transition corresponding to crystallization under the measurement conditions. Based on its amorphous diffraction pattern and glass transition behaviour, the synthesized **1** can be categorized as a CP glass.

The mechanical properties of **1** were evaluated from viscosity and dynamic mechanical analyses (DMA). The viscosity of **1** showed a higher value than that of **2** in the measurement temperature range (−20 to 120 °C) (Fig. 1C). The viscosity values of **1** are lower than the viscosity at the Littleton softening point (10^6.6^) and higher than that at the working point (10^4^).^28^ Soda lime glass exhibits these viscosities around 700–800 °C. The viscosity of **1** at room temperature to 120 °C is suitable for making a large transparent monolith by an annealing process (Fig. 1B; inset). Furthermore, DMA of **1** also demonstrated a higher shear modulus than that of **2** (Fig. S5†). The *G*′′ (the loss modulus) of **1** is lower than *G*′ (the storage modulus) at each temperature (−20 to 120 °C). This suggests that **1** has no fluidity even above the glass transition temperature.

**Fig. 2:**
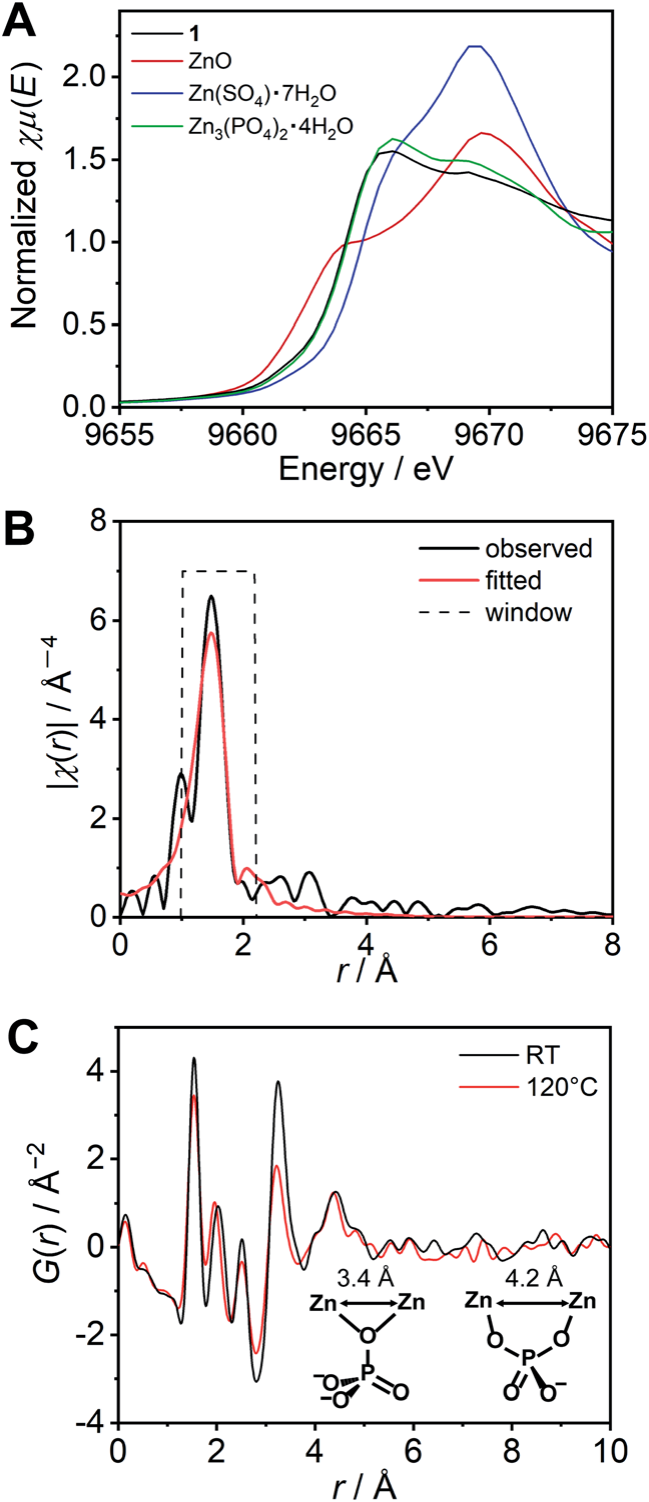
(A) The XANES region of the XAFS spectra for the Zn^2+^ K-edge of **1** (black) and references: ZnO (red), Zn(SO_4_)·7H_2_O (blue), and Zn_3_(PO_4_)_2_·4H_2_O (green). (B) Radial distribution functions (RDF) of **1** (black line) and the fitting curve (red line). (C) The reduced pair distribution function (PDF) profile of **1** and Zn^2+^–Zn^2+^ distances.

We subsequently characterized the amorphous structure of **1**. X-ray absorption fine structure (XAFS) analysis is often used for consideration of the coordination geometry and average coordination number of Zn^2+^.^29–31^ The XAFS spectra of the K-edge region of Zn^2+^ for **1** and the reference samples were measured and analysed (Fig. S6†). As references, three Zn^2+^ salts adopting different coordination geometries, *i.e.*, ZnO (tetrahedral), Zn(SO_4_)·7H_2_O (octahedral), and Zn_3_(PO_4_)_2_·4H_2_O (tetrahedral : octahedral = 2 : 1)^32^ were also measured. The edges of the spectra of the reference samples showed a relationship between the energy and the coordination number. The X-ray absorption near edge structure (XANES) region of **1** (Fig. 2A) exhibited a similar energy and shape to that of the model crystal Zn_3_(PO_4_)_2_·4H_2_O, which indicates that the averaged coordination number of **1** is in the range of 4–6, and close to 4.67 (tetrahedral : octahedral = 2 : 1). The resulting *k*^3^ weighted radial distribution function of **1** was fitted using the FEFF calculation (Fig. 2B). The obtained converged parameter gave a reasonable coordination number (4.6 ± 0.4), which is close to the proposed model Zn_3_(PO_4_)_2_·4H_2_O. The fitted coordination number of 4.6 can only be explained by some H_2_PO_4_^−^/H_3_PO_4_ coordinating more than two Zn^2+^ ions. These results suggest that H_2_PO_4_^−^ acts as a bridging ligand and that a Zn^2+^–H_2_PO_4_^−^/H_3_PO_4_ coordination network structure is formed in **1**.

The reduced pair distribution function (PDF), *G*(*r*), was conducted using a wide *Q* range diffraction measured at the synchrotron (incident energy = 61.377 keV). The *G*(*r*) of **1** exhibited five distinct peaks (at 1.54, 2.03, 2.51 3.25, and 4.42 Å) observed below 5 Å (Fig. 2C). A related crystal structure of zinc phosphate (Zn_3_(PO_4_)_2_·4H_2_O)^32^ was used to consider the possible Zn^2+^–Zn^2+^ distances in the Zn^2+^–H_2_PO_4_^−^/H_3_PO_4_ network structure. In the model crystal structure, two types of Zn^2+^–Zn^2+^ distances were found to be shorter than 5 Å. The shortest Zn^2+^–Zn^2+^ distance (3.38 Å) was formed *via* one μ-oxygen atom of the PO_4_^3−^ anion. Another Zn^2+^–Zn^2+^ distance (4.22 Å) bridged by two oxygen atoms of a PO_4_^3−^ was also found (Fig. 2C inset). The peaks obtained in the PDF are in good agreement with the two aforementioned Zn^2+^–Zn^2+^ distances. In addition to the comparison with the model crystal, the atomic scattering factor of Zn^2+^ is much higher than that of the other atoms. Therefore, the two peaks (3.25 and 4.42 Å) can be assigned to Zn^2+^–Zn^2+^. The PDF measured at 120 °C also showed similar peaks. The Zn^2+^–Zn^2+^ correlations are retained at high temperatures, even the intensity of the peak at 3.3 Å is slightly lower than that obtained at room temperature. The XAFS and PDF analyses of **1** suggest the formation of coordination networks.

**Fig. 3:**
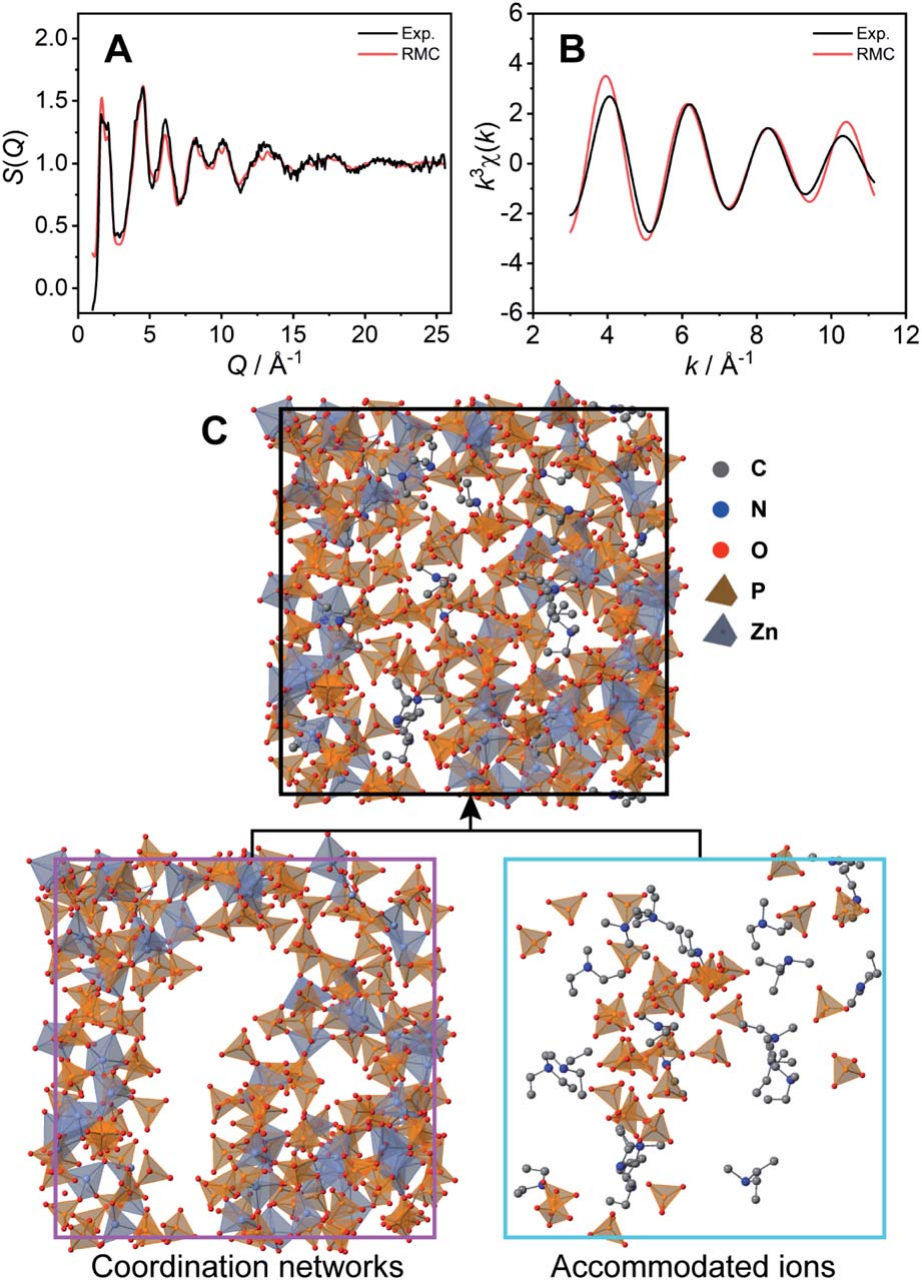
Comparison of the experimental data (black lines) and the results of the RMC structural modelling (red lines) for **1**. (A) X-ray total structure factor *S*(*Q*) and (B) EXAFS *k*^3^*χ*(*k*). (C) The unit cell of the amorphous structure of **1** modelled by RMC. The coordination network and captured free dema and H_2_PO_4_^−^/H_3_PO_4_ are separately presented.

The coordination network structure was demonstrated by reverse Monte Carlo (RMC) structural modelling using the X-ray experimental structural factor *S*(*Q*) and EXAFS *k*^3^*χ*(*k*) data at room temperature with several coordination number constraints. In this simulation, the atoms (Zn: 60, P: 180, O: 720, N: 21, C: 105, and H: 693) were placed in a cubic box. The size of the cubic box was determined by the experimental density (1.87 g cm^−3^) at room temperature. The calculated *S*(*Q*) (Fig. 3A) and *k*^3^*χ*(*k*) (Fig. 3B) of the RMC structural modelling indicated good agreement with the experimental values. The resulting RMC modelling structure of a cubic unit cell is presented in Fig. 3C (Table S2†). The averaged coordination number of Zn^2+^ is 4.4, which is close to that obtained by the XAFS measurements (4.6).

The obtained CP structure contains all 60 Zn^2+^ atoms and 147 H_2_PO_4_^−^/H_3_PO_4_ molecules (Fig. 3C bottom left). In the structure, dema and free H_2_PO_4_^−^/H_3_PO_4_ are found in the voids of the three-dimensional coordination network (Fig. 3C bottom right). The RMC modelling structure suggests that bulky dema and free H_2_PO_4_^−^/H_3_PO_4_ are surrounded by coordination networks. The structure can play the role of exclusive proton conductivity with a low energy barrier.

**Fig. 4:**
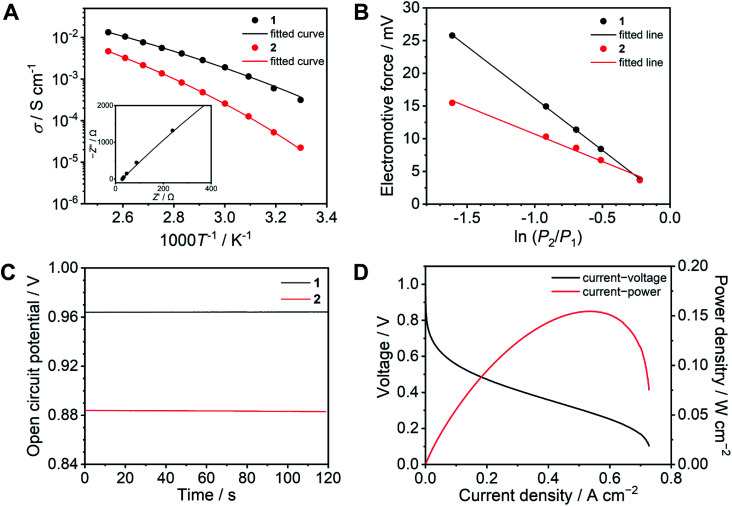
Proton conducting properties of **1** (black) and **2** (red). (A) Ion conductivity as a function of temperature under anhydrous conditions. The solid lines are fitting curves determined using the VFT equation (eqn (1)). The inset exhibits the Nyquist plots of **1** at 120 °C. (B) Electromotive force measured under different relative hydrogen gas pressure. (C) OCV of a H_2_/O_2_ fuel cell at 120 °C without humidification. (D) *I*–*V* (black) and *I*–*W* (red) curves of a H_2_/O_2_ fuel cell with **1** as an electrolyte at 120 °C without humidification.

The ion conductivity of **1** was determined by variable temperature AC impedance spectroscopy under a dry inert atmosphere (Fig. 4A and S7†). The Nyquist plots at 120 °C are shown with fitted curves from an equivalent circuit model (Fig. S8†). The equivalent circuit contains R1, R2, and Q1, where R1 is the bulk resistance of **1** or **2**, and the parallel connection of R2 and Q1 represents combined resistance and constant phase elements from the electric double layer, respectively (Fig. S9 and S10†).^33^ The conductivity reached *σ* = 13.3 mS cm^−1^ at 120 °C, which is approximately twice as high as that of **2** (*σ* = 6.5 mS cm^−1^).^17^ In protic ionic liquids, the lower viscosity results in higher conductivity due to the contribution of the vehicle mechanism.^18^ The conductivities of **1** are higher than that of **2** at each temperature, whereas the viscosity of **1** (log *η*^−1^ = −5.6 P^−1^ at 120 °C) is significantly higher than that of **2** (log *η*^−1^ = −0.57 P^−1^ at 120 °C). This suggests that in comparison to **2**, the proton-conducting mechanism of **1** is dominated more by the Grotthuss mechanism. The temperature dependence of both **1** and **2** showed non-Arrhenius type plots which are frequently seen in ionic liquids and their polymer composites.^25,34^ These temperature profiles were analysed by curve fitting using the Vogel–Fulcher–Tammann (VFT) equation (eqn (1), see ESI† for details) to obtain the parameters for proton conductivity.^35,36^1
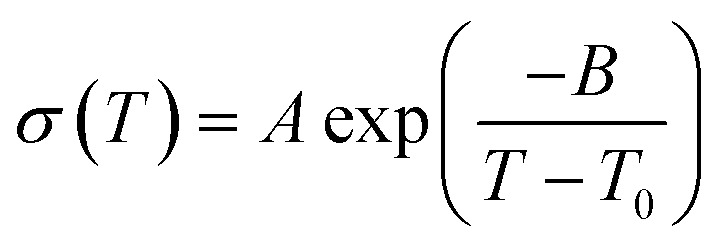


The resulting fitting parameters are in good agreement with the experimental results (Fig. 4A, solid lines) and are summarized in Table S1.† The smaller *A* value of **1** than that of **2** suggests that the concentration of conducting ions does not contribute to the higher conductivity. The higher conductivity of **1** is attributed to the lower pseudo activation energy (*B*). This suggests that the coordination network provides a better proton-conducting path than fluidic liquid states.

To obtain further insight, electrochemical measurements were subsequently carried out in a H_2_/O_2_ fuel cell. The transport number is defined as the ratio of the electric current derived from an ion of interest or target ion to the total electric current.^37^ In the case of protic ionic liquids, a transport number is usually in the range of 0.5–0.6 which means that the counter anions contribute to the ion conduction.^17,38–40^ The transport numbers of **1** and **2** were determined by electromotive force (EMF) measurements between different hydrogen partial pressure values (−ln(*P*_2_/*P*_1_) = 0.22, 0.51, 0.69, 0.92, and 1.61) at 120 °C (Fig. 4B) using eqn (2) (see ESI†).^41^ The resulting transport number of **1** is 0.94, which indicates that the ion conductivity of **1** is predominantly attributed to the protons. On the other hand, the obtained transport number for **2** is 0.49. The high transport number of **1** suggests that the movement of anions is significantly suppressed by coordination bond formation with Zn^2+^. To confirm the impact of the high transport number of **1**, we measured the OCV in a H_2_/O_2_ fuel cell at 120 °C (Fig. 4C). The OCV of **1** is 0.96 V and it is intact over 10 minutes, it is also higher than that of **2** (0.88 V), while the observed OCV of **1** is lower than the theoretical value of 1.16 V at 120 °C,^42^ it is comparative to the highest OCV values of CP/MOFs and protic ionic liquids at 120 °C under anhydrous conditions reported so far.^23,43,44^

The *I*–*V* curve of the H_2_/O_2_ fuel cell with **1** was determined at 120 °C under anhydrous conditions (Fig. 4D). The electrolyte membrane was prepared by impregnation of **1** into a polytetrafluoroethylene (PTFE) membrane. The *I*–*V* curve was measured using 100% H_2_ and O_2_ gases. We also attempted to evaluate the *I*–*V* curve for **2**, however, this was unsuccessful under the same conditions. This is because of high fluidity and because it is hard to suppress the gas permeation using **2** as the electrolyte. The observed maximum power density was 150 mW cm^−2^. Among the fuel cell performance in related conditions at 120 °C, this value is higher than that of the reported CP/MOFs,^44,45^ xerogels,^46^ covalent organic frameworks (COFs),^7,47^ and membrane composite of protic ionic liquids^21,40^ and as high as the composite of functionalized graphene oxide and Nafion (150 mW cm^−2^) measured under humidified conditions (RH = 25%).^48^ Furthermore, neat **1** was tested as an electrolyte layer without the PTFE membrane (Fig. S11†). The OCV value of the fuel cell with neat **1** exhibited almost the same value (0.96 V). This suggests that the CP glass can be used directly as an electrolyte, although the OCV stability during measurement (10 min) is slightly lower than that measured with the PTFE membrane.

**Fig. 5:**
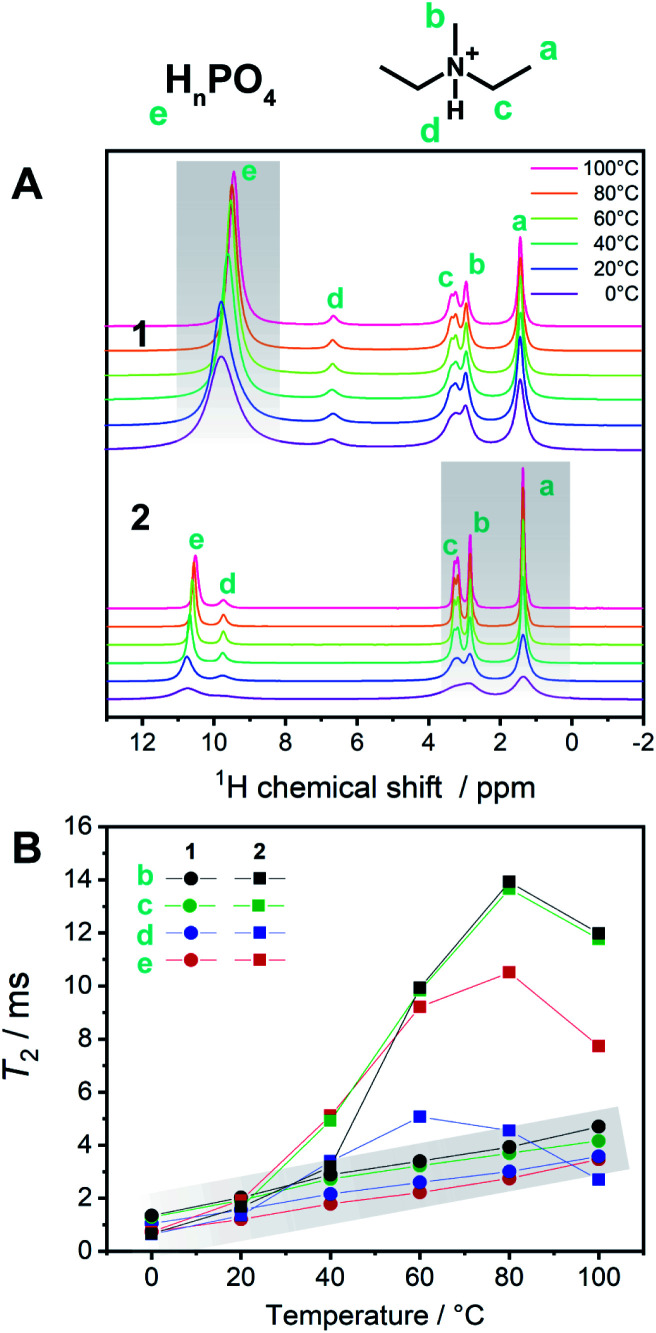
(A) ^1^H SSNMR spectra of **1** and **2** from 0 to 100 °C including the assignments of protons. Narrowing of phosphates peaks in **1** upon heating is highlighted. (B) *T*_2_ of ^1^H SSNMR signals of **1** (circles, gray highlight) and **2** (squares) at each temperature.

To describe the dynamics of the coordination network and protons in **1**, we carried out ^1^H SSNMR from 0 to 100 °C for **1** and **2** (Fig. 5A). The chemical shifts of the protons on the aliphatic carbons of dema (4–1 ppm) were independent of the temperature and were identical for **1** and **2**. On the other hand, the proton signals of H_2_PO_4_^−^/H_3_PO_4_ were observed at 10–9.5 (**1**) and 11–10.4 (**2**) ppm, respectively. Hydrogen bond formation has been reported in protic ionic liquids containing proton-donating and accepting anions such as HSO_4_^−^ and H_2_PO_4_^−^.^19,23^ When the hydrogen bonds are formed in liquid states or solutions, downfield shifts are often observed.^16^ A dimer formation *via* two hydrogen bonds in H_2_PO_4_^−^ is formed and these protons trapped by the dimer structure are not favourable for proton conduction. The presence of Zn^2+^ decreases the hydrogen bonds between H_2_PO_4_^−^/H_3_PO_4_ by forming a coordination network. Considering the temperature dependence of **1**, only the acidic proton exhibited a relatively high dependence of the chemical shifts and peak broadening on temperature, whereas both the cations and anions of **2** showed temperature-dependent broadening. This difference suggests that the conductivity of **1** is mainly affected by the proton of H_2_PO_4_^−^/H_3_PO_4_, which is in good agreement with other measurements. It was reported that the ion conductivities of protic ionic liquids are related not only to the protons but also to the cations and anions.^23^ The observed spin–spin relaxation time (*T*_2_) of **2** is much longer than that of **1** in the temperature region of 0–100 °C (Fig. 5B). The shorter *T*_2_ for **1** means that it exhibits more solid-like behaviour, and **2** is in a more isotropic fluid state.

These SSNMR analyses support the observation that the coordination networks in **1** enhance the conductivity and increase the proton transport number.

## Conclusions

In conclusion, we propose an approach for creating a CP glass from a protic ionic liquid for solid-state anhydrous proton conductors. The reaction of Zn^2+^ and protic ionic liquid (dema)(H_2_PO_4_^−^) formed a Zn^2+^–H_2_PO_4_^−^/H_3_PO_4_ network structure, and dema cations were surrounded by this CP network. The synthesized compound **1** is a glassy solid exhibiting sufficient mechanical strength to form a membrane. The amorphous structure was characterized by XAFS and PDF, which indicates the formation of a coordination network. The three-dimensional coordination polymer structure was proposed by RMC modeling, which was in good agreement with the experimental data. The formation of the extended networks prohibits the movement of both cations (dema) and anions (H_2_PO_4_^−^). This coordination driven structure prevents formation of hydrogen bonds, and the glassy solid **1** shows higher proton conductivity (13.3 mS cm^−1^) than liquid **2** at 120 °C. Consequently, we observed exclusive proton conduction in the structure. This is confirmed by the high transport number (0.94) and OCV (0.96 V). These proton-conducting and viscoelastic properties of **1** demonstrated the highest performance in a H_2_/O_2_ fuel cell (0.15 W cm^−2^) among the reported CP/MOF conductors. The characteristic proton-conducting properties and structural analyses indicate that the coordination network formation from protic ionic liquids is promising for the modification of mechanical properties and proton conductivity.

## Conflicts of interest

There are no conflicts to declare.

## Supplementary Material

SC-011-D0SC01737J-s001
