# Supplementary material for: Coordination polymer glass from a protic ionic liquid: proton conductivity and mechanical properties as an electrolyte
Source: Chem Sci. 2020 Apr 17;11(20):5175–81. doi: 10.1039/d0sc01737j (PMC8159284; doi:10.1039/d0sc01737j)
Supplement: SC-011-D0SC01737J-s001 [file SC-011-D0SC01737J-s001.pdf]

Electronic Supplementary Material (ESI) for Chemical Science.
This journal is © The Royal Society of Chemistry 2020

Supporting Information for

Coordination Polymer Glass from Protic Ionic Liquid: Proton Conductivity and Mechanical Property as Electrolyte

Tomohiro Ogawa,[a,b] Kazuki Takahashi,[c] Sanjog S. Nagarkar,[a] Koji Ohara,[d] You-lee Hong,[b,e] Yusuke Nishiyama,[e,f] and Satoshi Horike*[a,b,g,h]

[a]AIST-Kyoto University Chemical Energy Materials Open Innovation Laboratory (ChEM-OIL), National Institute of Advanced Industrial Science and Technology (AIST), Yoshida-Honmachi, Sakyo-ku, Kyoto 606-8501, Japan

[b]Institute for Integrated Cell-Material Sciences, Institute for Advanced Study, Kyoto University, Yoshida-Honmachi, Sakyo-ku, Kyoto 606-8501, Japan

[c]Advanced Research and Innovation Center, DENSO CORPORATION, 500-1 Minamiyama, Komenoki-cho, Nisshin, Aichi 470-0111, Japan

[d]Diffraction and Scattering Division, Center for Synchrotron Radiation Research, Japan Synchrotron Radiation Research Institute (JASRI), Kouto, Sayo, Hyogo 679-5198, Japan

[e]RIKEN-JEOL Collaboration Center, Yokohama, Kanagawa 230-0045, Japan

[f]NMR Science and Development Division, RIKEN SPring-8 Center, Yokohama, Kanagawa 230-0045, Japan, and JEOL RESONANCE Inc., Akishima, Tokyo 196-8558, Japan

[g]Department of Synthetic Chemistry and Biological Chemistry, Graduate School of Engineering, Kyoto University, Katsura, Nishikyo-ku, Kyoto 615-8510, Japan

[h]Department of Materials Science and Engineering, School of Molecular Science and Engineering, Vidyasirimedhi Institute of Science and Technology, Rayong, 21210, Thailand

Table of contents

Experimental

Scheme S1. Synthetic scheme of **1** and **2**.

Table S1. VFT fitting parameters of H^+ conductivity.

Fig. S1. Solid-state ^1H NMR spectra of **1** and **2** at 20 °C.

Fig. S2. Solid-state ^{31}P NMR spectra of **1** and **2**.

Fig. S3. IR spectra of **1** and **2**.

Fig. S4. TGA and DSC curves of **1** under inert atmosphere.

Fig. S5. DMA of **1** and **2** from −20 °C to 120 °C.

Fig. S6. XAFS spectra of Zn K-edge of **1** and references.

Fig. S7. Nyquist plots of **1** and **2**.

Fig. S8. Nyquist plots and fitting curve of **1** and **2** at 120 °C.

Fig. S9. Equivalent circuit for the fitting of impedance measurements.

Fig. S10. Bode plots for the impedance measurements and an equivalent circuit model.

Fig. S11. OCV of a H_2/O_2 fuel cell using **1** as an electrolyte without PTFE membrane.

Table S2. Simulated RMC structure in cif format.

References

EXPERIMENTAL

Materials and methods: ZnO (99.99% trace metals basis) and diethylmethylamine were purchased from Aldrich, and phosphoric acid (85% in H_2O) was purchased from Wako pure chemical industries. All reagents were used without further purification process. The reported ionic liquid (dema)(H_2PO_4) (**2**) was synthesized according to the literature method.[1]

Synthesis: Synthesis of Zn-dema-H_2PO_4 (**1**): ZnO (3.25 g, 0.04 mol) and 3 equimolar of phosphoric acid (85%) (8.18 mL, 0.12 mol) were mixed and grinded in a mortar until form transparent colorless liquid, then, excess H_3PO_4 was neutralized by addition of 1 equimolar diethylmethylamine (4.84 mL, 0.04 mol) and grinding for 30 min. Then resulting transparent colorless liquid was transferred to a round bottom flask and dried under reduced pressure at room temperature for 8 h and then at 120 °C for 2h. After cooling to room temperature, transparent high viscoelastic colorless material was obtained (13.9 g). The chemical composition of **1** was determined by solid-state NMR and elemental analysis. $(dema)_{0.35}[Zn(H_2PO_4)_{2.35}(H_3PO_4)_{0.65}]$. Elemental analysis calcd for $C_{1.75}H_{11.55}N_{0.35}O_{12}P_3Zn$: C, 5.42; H, 3.00; N, 1.26, found C, 5.64; H, 3.36; N, 1.30. Density = 1.87 g cm^{-3}.

Thermogravimetric analysis (TGA) were recorded using Rigaku TG8120 instrument under flowing N_2 with 10 K min-1 scan rate. Differential Scanning Calorimetry (DSC) experiments were measured using Hitachi DSC 7020 instrument in an inert argon atmosphere. The sample was placed in an aluminum sample folder and a lid.

Impedance spectra from 30 °C to 120 °C under Ar atmosphere were collected using a BioLogic VSP-300 with an EC Frontier sample cell (SB1A, ϕ = 13 mm, d = 5 mm). The frequency ranges from 0.1 Hz to 1.0 MHz with a sinus voltage (10 mV) was collected at each temperature. The measurements were performed at thermal equilibrium after holding 30 min at each temperature. Collected impedance data were treated and analyzed by a ZView software. The VFT (eq. 1) fitting (Levenberg-Marquardt Method) was performed using an Origin 2018 software.

$$\sigma(T) = A\,exp(\frac{-B}{T-T_0}) \qquad \text{(eq. 1)}$$

A is proportional to the concentration of the carrier ions, B is the pseudo activation energy for the ion conduction, while T_0 is the ideal glass transition temperature.

Proton Transport number (t_{H+}) was determined by the electromotive force (EMF) measurements using a BioLogic VSP-300. A membrane filter (Omnipore™ Merck, 10.0 uM, 2 X 2 cm) was impregnated with **1** or **2** at 120 ºC or at room temperature, respectively. The membrane was then sandwiched between Pt loaded carbon papers (1.5 mg cm^{-2}, ϕ = 5 mm, Chemix Co. Ltd.) and placed into a single cell with straight gas flow channels. On one side H_2 /Ar (3.99 vol%,) was fed continuously while on the other side the H_2/Ar gas was diluted with N_2 (99.99995 vol%) gas to achieve variable partial pressures. The H_2 concentration was precisely controlled by mass flow controllers (SEC-E40, Horiba, ltd.) while maintaining total gas flow rate of 100 mL/min on both the sides. The assembly was maintained at desired temperature using Oven and stabilized for ~10 min before measurement. The EMF generated between the sensing and counter electrodes were measured under different H_2 partial pressures (P_2) on equilibrating for 3 min. The proton transport number (t_{H+}) is calculated using following equation

$$E = t\frac{RT}{2F}\ln\left(\frac{P_1}{P_2}\right) \quad \text{(eq. 2)}$$

where, E, T, R, F, P_1, P_2, and t denote EMF, temperature, the gas constant, Faraday constant, partial pressure of H_2 gas, and transport number, respectively.

The polarization curves of a H_2/O_2 fuel cell were recorded using a Solartron SI 1287. Gas diffusion electrodes (0.3 mg/cm^2, 46.5 wt% Pt/C) for both cathode and anode were fabricated by spray coating of 46.5wt% Pt/C (TEC10E50E, Tanaka Kikinzoku, ltd.) onto the carbon paper gas diffusion layer (Sigracet® 29 BC, SGL Carbon, GmbH.). A membrane electrode assembly (MEA) was prepared by sandwiching the electrolyte membrane between two gas diffusion electrodes, and the contact area between electrode and electrolyte were ϕ = 7 mm. The resulting MEA was installed into a single cell with straight gas flow channels, and the dry H_2 (>99.999vol%) and O_2 (>99.999vol%) gases were supplied to the single cell at a flow rate of both 100mL/min under anhydrous conditions. The flow rate was controlled by mass flow controllers (SEC-E40, Horiba, ltd.). The polarization curves of the single cell were measured at 120 °C.

Extended X-ray Absorption Fine-Structure Spectroscopy were collected on beamline BL14B2 at SPring-8. X-ray absorption spectra in the energy region of the Zn K-edge were measured in transmission mode. Fourier transformation was k3-weighted in the k range from 3.0 to 12.5 $Å^{-1}$. The data processing and coordination number fitting were performed with Athena and Artemis software, respectively. All samples were measured under Ar atmosphere. The resulting k3-weighted radial distribution function of **1** was fitted by a FEFF calculation using first Zn-O single scattering path of $Zn(SO_4)\cdot 7H_2O$.

X-ray total scattering data were collected with four CdTe and two Ge detectors covering the Q range up to 25 $Å^{-1}$ at SPring-8 on beamline BL04B2 (61.377 keV; λ = 0.2020 Å). The collected scattering data was applied absorption, background, and Compton scattering corrections, and then normalized to give the Faber−Ziman total structure factor $S(Q)$.[2,3] Then, the pair distribution function was calculated by Fourier transforming the $S(Q)$ with a Lorch modification function.[4]

Reverse Monte Carlo structural modeling of amorphous state of **1** (a cubic cell; $a = b = c = 27.44$ Å) was performed using the RMC++ program.[5] The initial structure was generated by random command. The initial configuration was converted by hard-sphere Monte Carlo simulations with constraints applied to avoid unrealistic structures. The closest distance, bond angles and bond length constraints ware performed to form reasonable diethylmethylammonium cations and $H_2PO_4^-$/H_3PO_4 structures (the constraints were listed below). The closest atom–atom distance was chosen based on the information from related crystal structures of TBA(H_2PO_4) and $Zn_3(PO_4)_2 \cdot 4H_2O$. After the hard-sphere Monte Carlo simulations, RMC simulations containing X-ray $S(Q)$ and $k^3\chi(k)$ EXAFS data were performed with the constraint of averaged coordination number of Zn-O = 4.6. The EXAFS back scattering tables were prepared by the FEFF[6] calculations. The reported atomic scattering factors and anomalous scattering factors of each atoms were used for these calculations. The protons were put using constraints and RMC simulations, although the X-ray $S(Q)$ experimental data does not include much information from protons. Therefore, the position of protons from RMC structure was not used for discussion of proton conducting mechanism.

The list of constraints applied in RMC structural modeling is listed as follows

Bond constraints	distance (Å)	Angle constraints	degree (°)
N-C	1.45-1.60	C-N-C	110
C-C	1.45-1.60	C-C-N	110
C-H	0.80-1.00	C-N-H	110
N-H	0.80-1.00	C-C-H	110
P-O	1.45-1.70	H-C-H	110
O-H	0.80-1.00	O-P-O	110
Zn-O	1.80-2.20	P-O-H	110
		O-Zn-O	105

Solid-state NMR experiments were performed on JNM-ECZ600R spectrometer (JEOL RESONANCE Inc., Japan) at 14.01 T with a ^{1}H resonance frequency of 599.7 MHz, equipped with a 2.0 mm double resonance MAS probe (JEOL RESONANCE Inc, Japan). 1D ^{1}H and ^{31}P spectra, and ^{1}H T_2 relaxation time were measured by single pulse and spin-echo pulse sequence, respectively, at 20 kHz spinning rate in the temperature range of 0-100 °C. RF field strength of ^{1}H and ^{31}P nuclei was 121.95 kHz at the $\pi/2$ pulse width of 1.98 μs and 131.58 kHz at the $\pi/2$ pulse width of 1.9 μs, respectively. The spectra were processed with the Delta software (JEOL RESONANCE Inc).

Laboratory scale powder X-ray radiation diffraction patterns were collected on a Rigaku SmartLab multipurpose diffractometer (CuKα; λ = 1.540598 Å).

FT-IR spectra were measured with a Thermo Scientific Nicolet Summit FT-IR equipped with a diamond ATR accessory.

Variable-temperature viscoelastic measurement was performed by using a rotational parallel-plate rheometer (Rheosol-G5000, UBM Co., Ltd.) under dry N_2 flow with applying oscillatory strain of 1 Hz. The sample-holding gap was set to 1.0 mm.

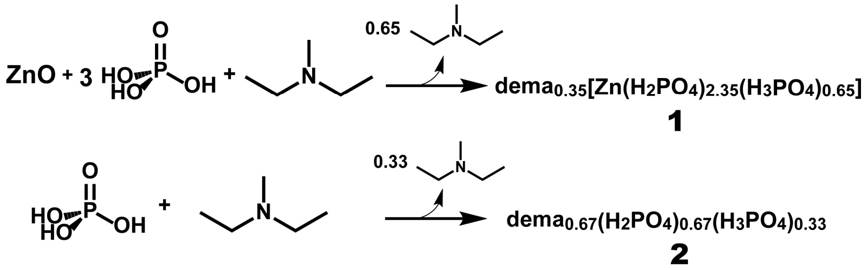

Scheme S1. Synthetic scheme of **1** (top) and **2** (bottom).

Table S1. VFT fitting parameters of H^+ conductivity using eq. 1.

	A (S cm^{-1})	B (K)	T_0 (K)	R^2
**1**	4.8 (±3.3)	1400 (±294)	155 (±21)	0.999
**2**	8.2 (±1.6)	1600 (±74)	179 (±4.4)	0.999

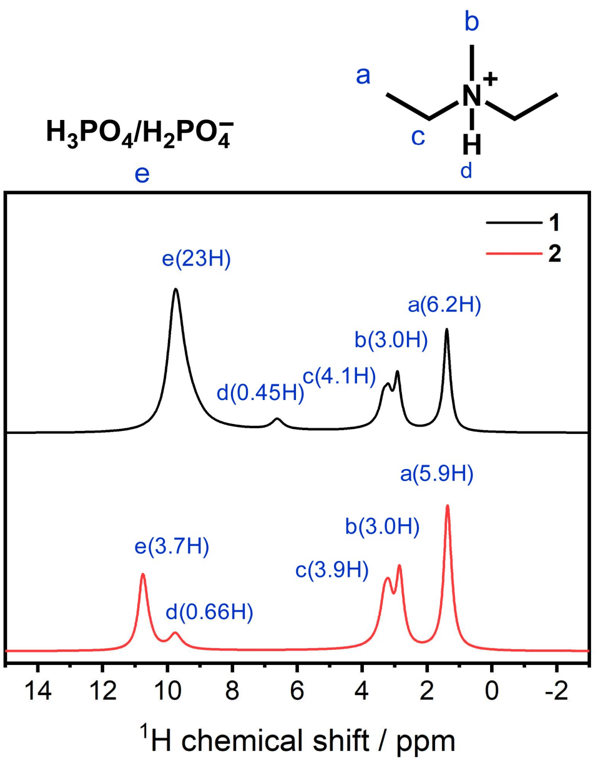

Fig. S1. Solid-state ^{1}H NMR spectra of **1** (black) and **2** (red) at 20 °C. The assignments and integration values are also shown.

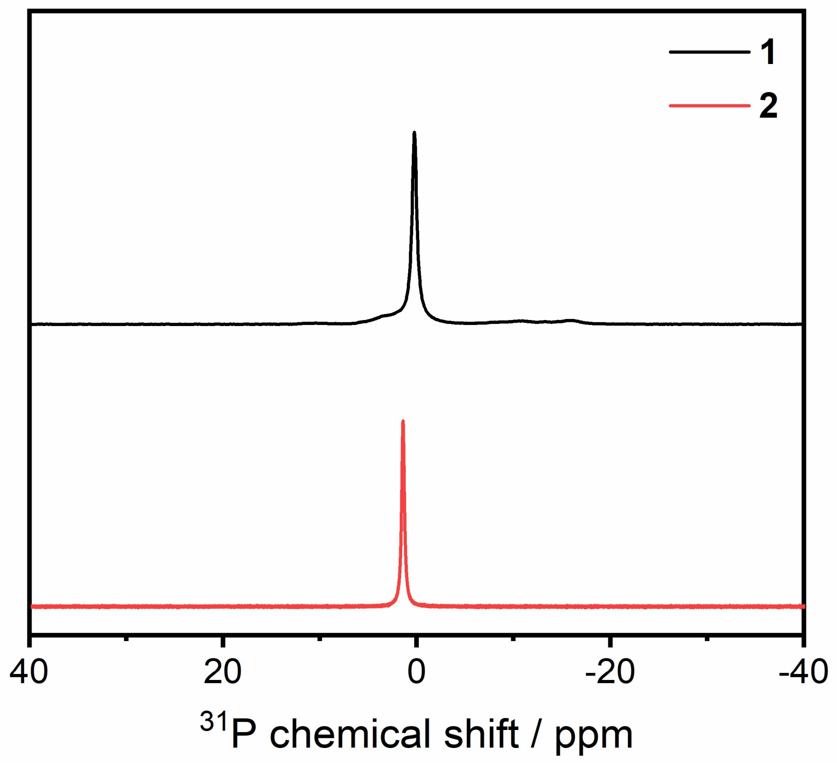

Fig. S2. Solid-state ^{31}P NMR spectra of **1** (black) and **2** (red) at 20 °C.

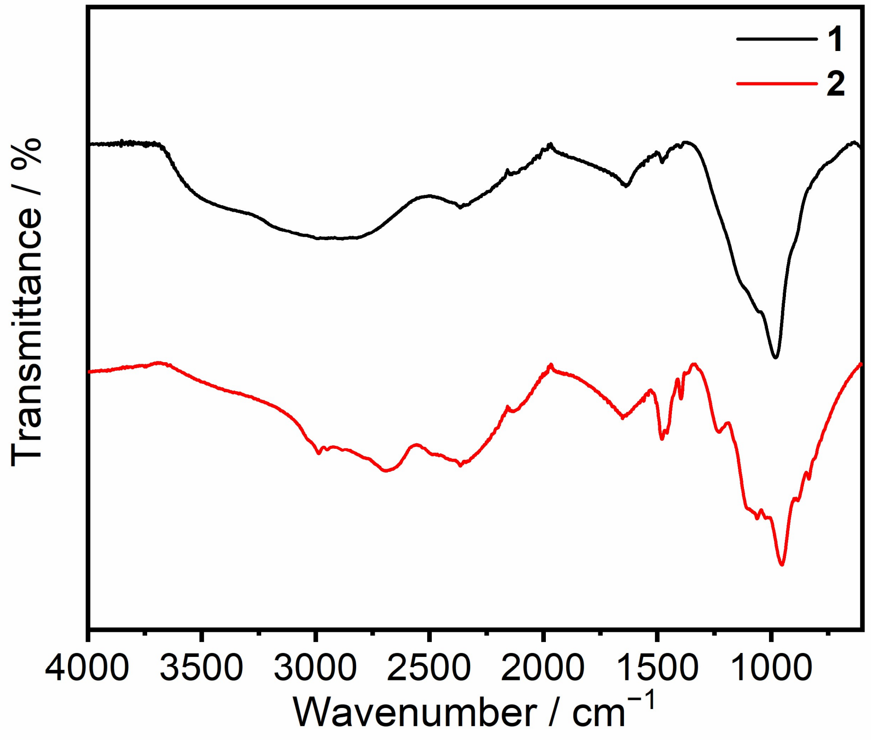

Fig. S3. IR spectra of **1** (black) and **2** (red) at room temperature.

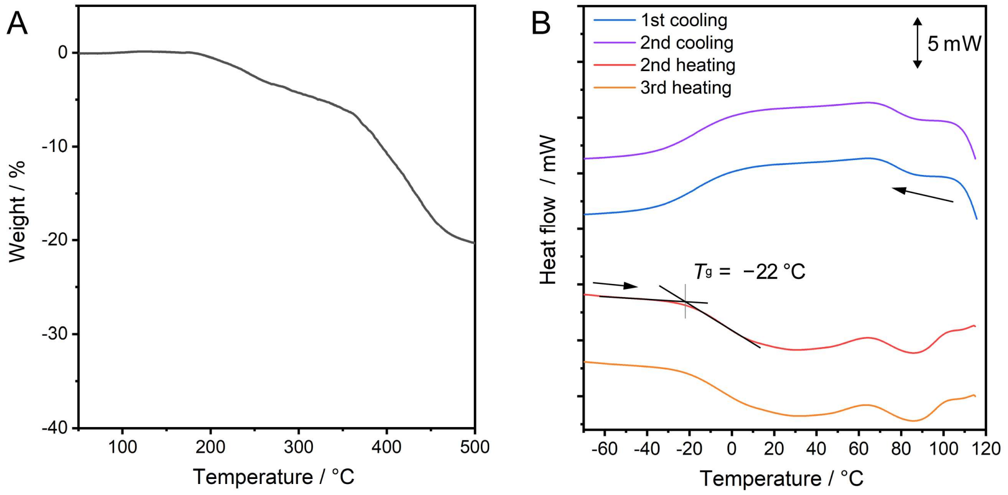

Fig. S4. (A) TGA curve of **1** under N_2 atmosphere (scan rate = 10 °C min^{-1}) and (B) DSC curves of first (blue) and second (purple) cooling, second (red) and third (orange) heating scans of **1** after initial first heating from room temperature under Ar atmosphere (scan rate = 10 °C min^{-1}). The viscoelastic **1** exhibited glass transition during cooling scan. Then, we determine the glass transition point in following heating scan (T_g = 22 °C). There is no peak assignable to crystallization up to 120 °C. However, continuous base line change around 60-100 °C cannot be assigned at this time. While the DMA properties at this temperature range exhibited similar complicated shape, this DSC baseline change is probably due to the change of coordination structures in Zn coordination polymer.

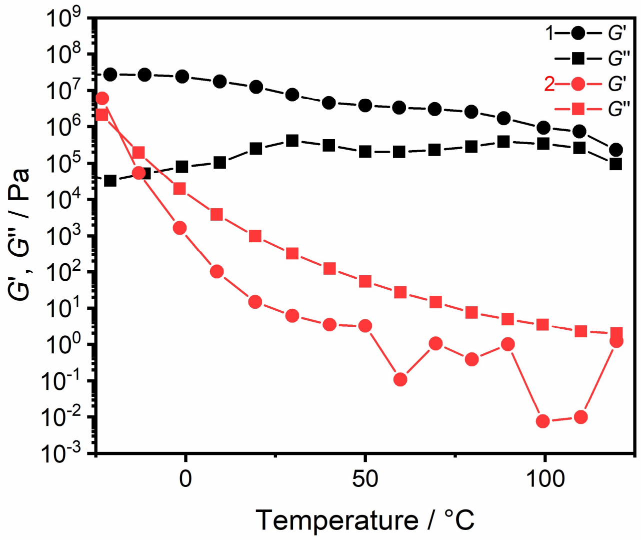

Fig. S5. DMA of **1** (black) and **2** (red) from −20 °C to 120 °C. The storage modulus and loss modulus were marked by circles (●) and squares (■), respectively.

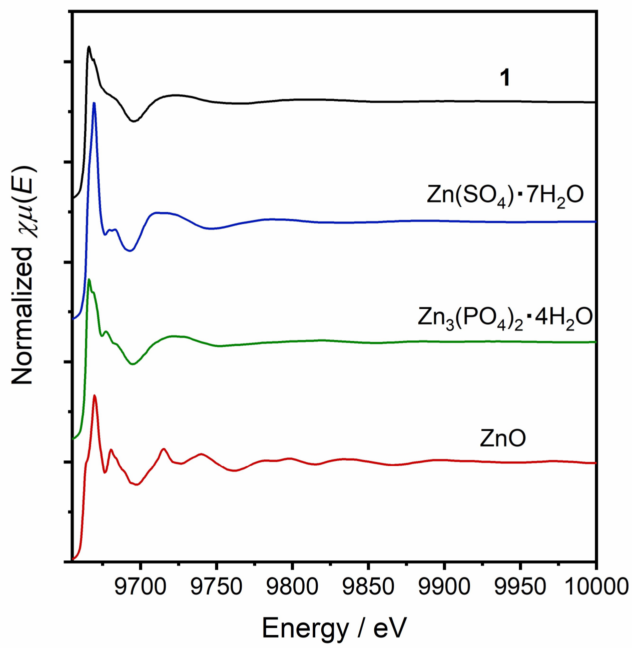

Fig. S6. XAFS spectra of Zn *K*-edge of **1** (black) and references with known coordination structures: $Zn(SO_4){\cdot}7H_2O$ (blue), $Zn_3(PO_4)_2{\cdot}4H_2O$ (green) and ZnO (red).

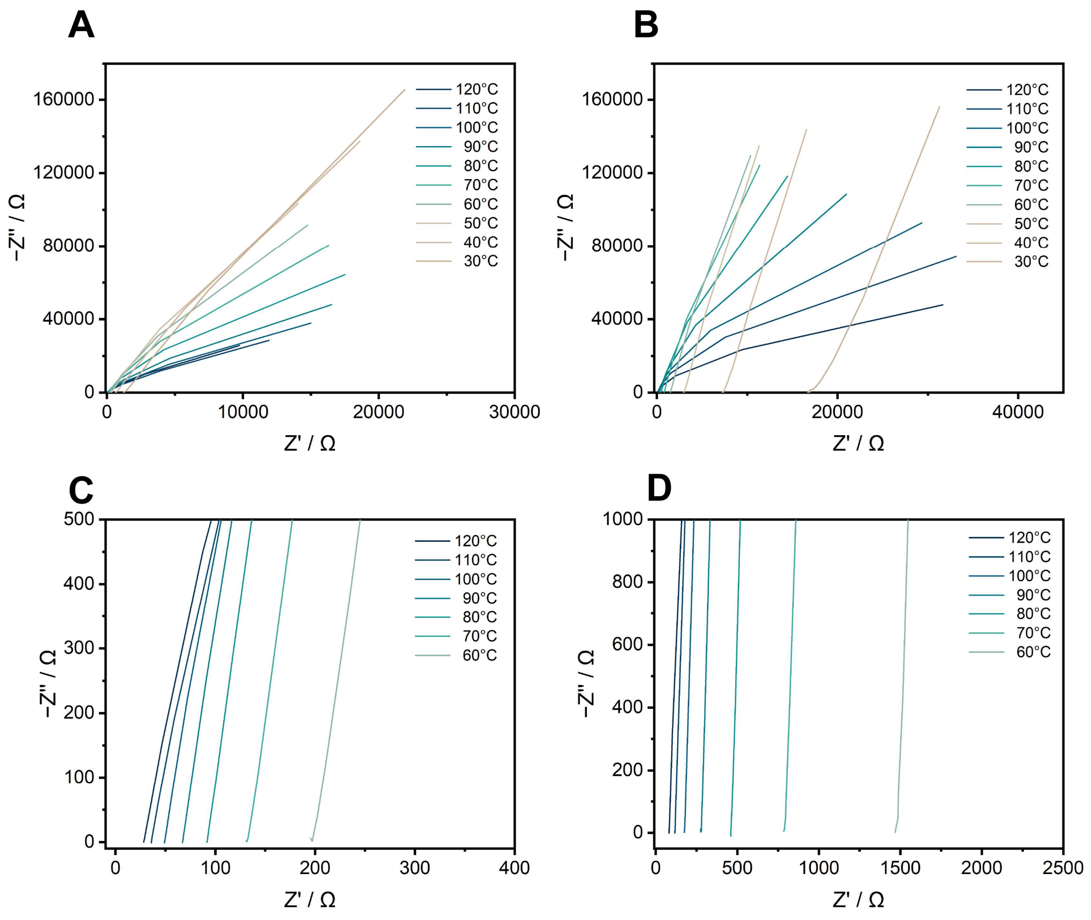

Fig. S7. Nyquist plots of (A) **1** and (B) **2** from 1.0 MHz to 0.2 Hz measured at 30-120 °C. The enlarged low resistance regions of (C) **1** and (D) **2** are also shown.

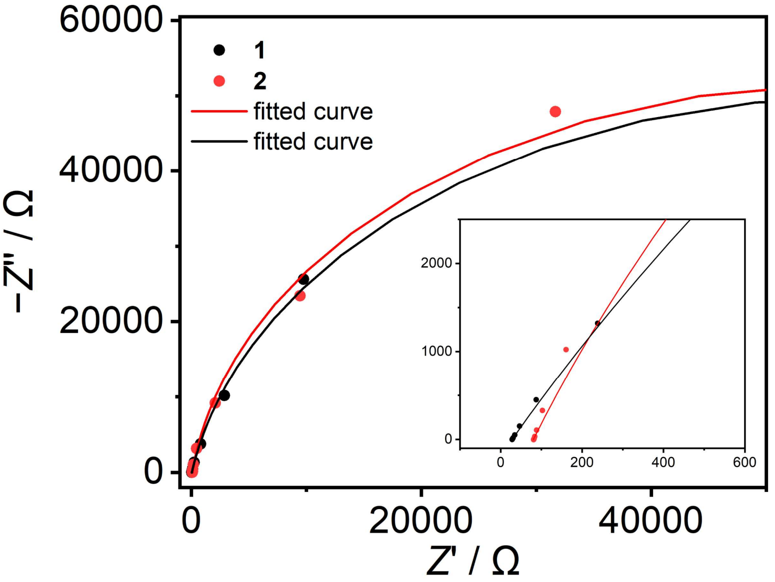

Fig. S8. Nyquist plots of **1** (black) and **2** (red) at 120 °C. The solid lines represent the fitted curves obtained by an equivalent circuit model (Fig. S8). Inset showed enlarged plot around low resistance region.

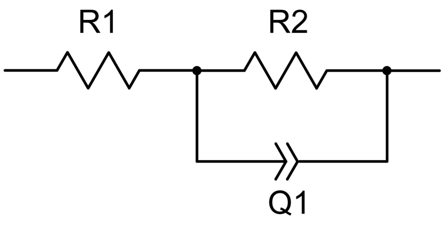

Fig. S9. Equivalent circuit for the fitting of impedance measurements. R1, R2 and Q1 represent the bulk resistance of **1** or **2**, resistance and constant phase element of the interface between electrodes and electrolytes, respectively.

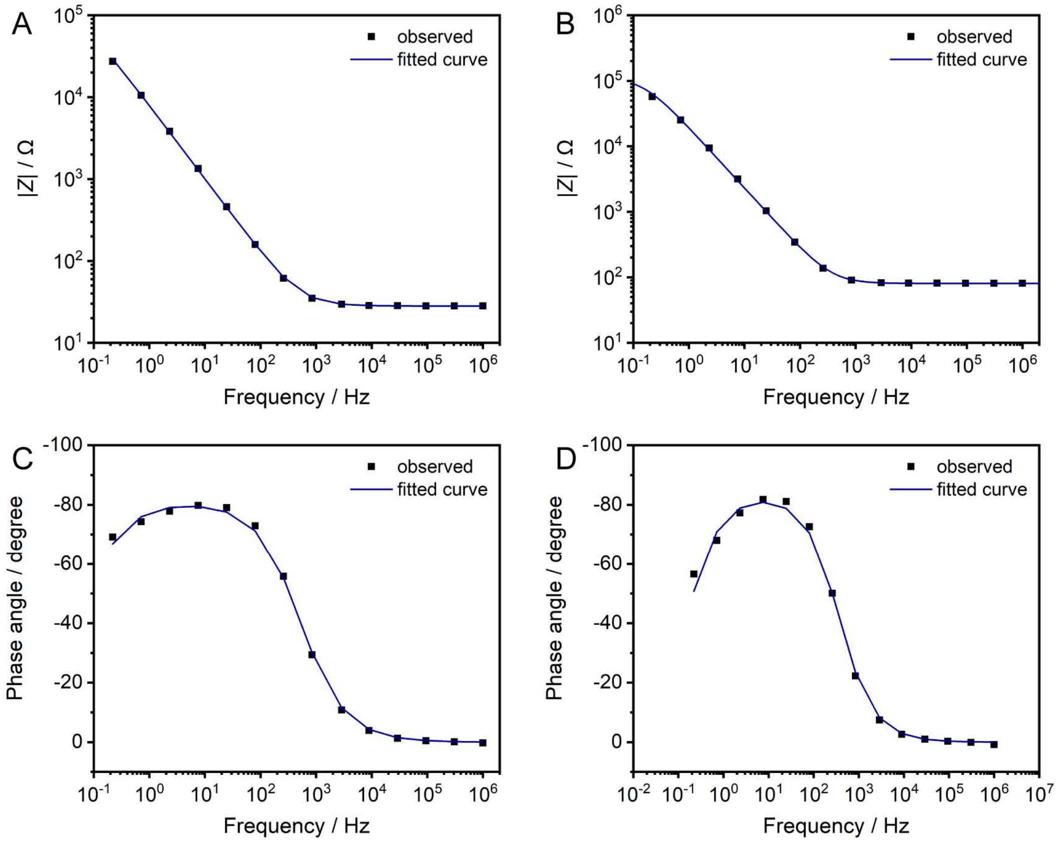

Fig. S10. Bode plots for the impedance measurements. |*Z*| vs frequency plots for (A) **1** and (B) **2**. Impedance phase angle vs frequency plots for (C) **1** and (D) **2**. The fitted curves are plotted as blue lines by an equivalent circuit model shown in Fig. S8.

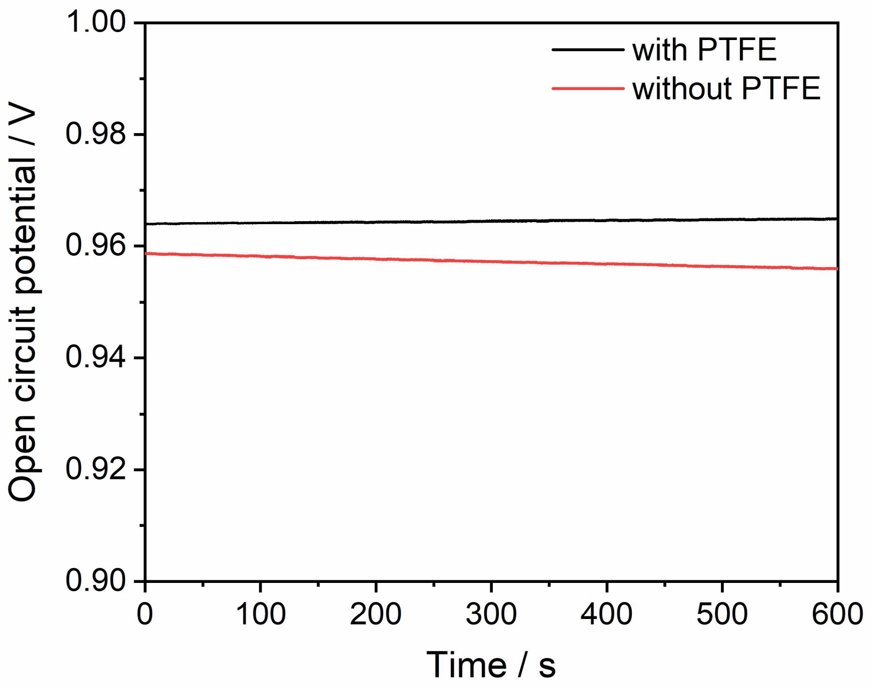

Fig. S11. OCV of a H_2/O_2 fuel cell using **1** as an electrolyte with PTFE (black) or without PTFE (red) at 120 °C without humidification.

Table S2. Simulated RMC structure in cif format. This text file can be opened in a visualization software for crystal structures.

_chemical_formula_sum	'Zn'
_cell_length_a	27.439582
_cell_length_b	27.439582
_cell_length_c	27.439582
_cell_angle_alpha	90
_cell_angle_beta	90
_cell_angle_gamma	90
_cell_volume	20660.10259
_cell_formula_units_Z	1
_symmetry_space_group_name_H-M	'P 1'
_symmetry_Int_Tables_number	1
_symmetry_cell_setting	cubic

loop_

_symmetry_equiv_pos_site_id

_symmetry_equiv_pos_as_xyz

1 'x, y, z'

loop_

_atom_site_label

_atom_site_type_symbol

_atom_site_fract_x

_atom_site_fract_y

_atom_site_fract_z

Zn1	Zn	0.122144652	0.212137494	0.742813302
Zn2	Zn	0.727756327	0.354130517	0.949666021
Zn3	Zn	0.586435851	0.281056544	0.017586505
Zn4	Zn	0.089533266	0.066108149	0.664246529
Zn5	Zn	0.364188533	0.27584323	0.675523167
Zn6	Zn	0.104697688	0.533546018	0.98704201
Zn7	Zn	0.360537487	0.92884936	0.483318385
Zn8	Zn	0.826574774	0.523406278	0.67935078
Zn9	Zn	0.155592688	0.90750379	0.515445687
Zn10	Zn	0.32621158	0.533524162	0.901464577
Zn11	Zn	0.187577379	0.357107852	0.902506243
Zn12	Zn	0.492505749	0.302964113	0.083862017
Zn13	Zn	0.614019846	0.33358989	0.92103056
Zn14	Zn	0.843573232	0.106746945	0.193532762
Zn15	Zn	0.647791101	0.074177955	0.779657399
Zn16	Zn	0.073088057	0.237611842	0.865249936
Zn17	Zn	0.678628977	0.164929699	0.897568355
Zn18	Zn	0.663587885	0.21782386	0.211157814
Zn19	Zn	0.459352923	0.885380548	0.220927982
Zn20	Zn	0.308687135	0.456712836	0.983529223
Zn21	Zn	0.577900215	0.225474742	0.910022766
Zn22	Zn	0.158563013	0.320812389	0.062592792
Zn23	Zn	0.402289716	0.966182023	0.005998519
Zn24	Zn	0.259787537	0.940104475	0.155705259
Zn25	Zn	0.441182228	0.596889595	0.777453082
Zn26	Zn	0.113090919	0.38115483	0.149483993
Zn27	Zn	0.83872315	0.686753628	0.766754384
Zn28	Zn	0.796629101	0.17073991	0.695080658
Zn29	Zn	0.208652158	0.234774931	0.008632305
Zn30	Zn	0.070926791	0.904201249	0.783457696
Zn31	Zn	0.838150189	0.974294087	0.546368531
Zn32	Zn	0.92089715	0.991524646	0.73277231
Zn33	Zn	0.773774894	0.254185302	0.550201373
Zn34	Zn	0.779025962	0.631168574	0.673774122
Zn35	Zn	0.407946545	0.706472451	0.759979395
Zn36	Zn	0.302397946	0.760441312	0.245718422
Zn37	Zn	0.70738843	0.531804312	0.009830302
Zn38	Zn	0.37181089	0.178818148	0.751980923
Zn39	Zn	0.054549032	0.223562066	0.529257719
Zn40	Zn	0.979636482	0.939475151	0.520462585
Zn41	Zn	0.505674087	0.783082556	0.242454701
Zn42	Zn	0.972523643	0.185592472	0.904457858
Zn43	Zn	0.250524181	0.824766977	0.893685813
Zn44	Zn	0.760728995	0.244212948	0.872603114
Zn45	Zn	0.41826996	0.136082203	0.531633674
Zn46	Zn	0.727935426	0.017030772	0.570508233
Zn47	Zn	0.036917795	0.795826908	0.068447279
Zn48	Zn	0.776167482	0.969537801	0.135636014
Zn49	Zn	0.254645419	0.951292055	0.333169623
Zn50	Zn	0.418903645	0.691247131	0.130989645
Zn51	Zn	0.663588819	0.578163264	0.816726837
Zn52	Zn	0.519741223	0.155637875	0.084673672
Zn53	Zn	0.344365307	0.394250048	0.550761771
Zn54	Zn	0.51495158	0.184199502	0.200164511
Zn55	Zn	0.362124785	0.827338965	0.329201797
Zn56	Zn	0.633226149	0.46966613	0.127583494
Zn57	Zn	0.016429585	0.047992418	0.493414978
Zn58	Zn	0.419336333	0.138029369	0.96528006
Zn59	Zn	0.473142574	0.036540535	0.160593632
Zn60	Zn	0.144356569	0.766829483	0.919600503
P1	P	0.598897705	0.718065417	0.275474427
P2	P	0.863926159	0.03963896	0.100161646
P3	P	0.310430059	0.501810436	0.49269793
P4	P	0.152251063	0.8390352	0.836932973
P5	P	0.122441202	0.676332547	0.548446924
P6	P	0.352023723	0.353178004	0.441532317
P7	P	0.472649829	0.294791474	0.687964669
P8	P	0.767682951	0.175264031	0.253318309
P9	P	0.077070957	0.493312488	0.551317845
P10	P	0.011916648	0.709755931	0.159039711
P11	P	0.364330408	0.645114395	0.853404402
P12	P	0.319392067	0.007161506	0.075476883
P13	P	0.390086375	0.36808302	0.936093252
P14	P	0.432951163	0.713288156	0.013143586
P15	P	0.892837129	0.720149262	0.863722085
P16	P	0.297716174	0.019372299	0.240613998
P17	P	0.620143186	0.478840861	0.333422259
P18	P	0.423084721	0.198428864	0.855456308
P19	P	0.225019237	0.259038351	0.133626413
P20	P	0.225554497	0.544499797	0.975498267
P21	P	0.894341747	0.583339172	0.746403032
P22	P	0.815611238	0.566188368	0.578214124
P23	P	0.429830641	0.499053226	0.845192623
P24	P	0.932094529	0.334135868	0.327740413
P25	P	0.015968302	0.074496116	0.130632556
P26	P	0.713327326	0.685731256	0.422621901
P27	P	0.274959149	0.86747807	0.261945877
P28	P	0.802051309	0.002605046	0.744643945
P29	P	0.717687165	0.321355948	0.314903563
P30	P	0.214251368	0.819121513	0.577943231
P31	P	0.716985617	0.615785699	0.095833213
P32	P	0.826506161	0.29546065	0.951730625
P33	P	0.672745838	0.864682237	0.899096623
P34	P	0.97370852	0.74075802	0.323270649
P35	P	0.143007957	0.287348734	0.395391771
P36	P	0.918635149	0.945366377	0.624610294
P37	P	0.540253998	0.313326693	0.831504977
P38	P	0.200038407	0.088443984	0.631239782
P39	P	0.3404641	0.386208474	0.666180416
P40	P	0.465570273	0.111487438	0.419518179
P41	P	0.505910539	0.990235476	0.264280302
P42	P	0.229823283	0.619840792	0.394735577
P43	P	0.446535936	0.628200897	0.664210111
P44	P	0.072451269	0.272815052	0.126547027
P45	P	0.901348574	0.06701059	0.492515819
P46	P	0.346924468	0.882155503	0.927480806
P47	P	0.515350798	0.691431789	0.534078136
P48	P	0.308977252	0.164452827	0.49956899
P49	P	0.565358716	0.637005035	0.769279519
P50	P	0.697730045	0.345566066	0.842926198
P51	P	0.930947216	0.76366713	0.024106056
P52	P	0.592962432	0.964812097	0.805908451
P53	P	0.663522117	0.022566571	0.672189973
P54	P	0.179106585	0.189801604	0.835680041
P55	P	0.042410855	0.91632918	0.028055538
P56	P	0.717609203	0.107184059	0.421079372
P57	P	0.785219069	0.851920138	0.775737043
P58	P	0.143889572	0.332537105	0.791657092
P59	P	0.020146875	0.176409888	0.791703914
P60	P	0.387428861	0.532134792	0.995482649

P61	P	0.301143661	0.687337745	0.520199193	P136	P	0.305819086	0.171403124	0.999143897
P62	P	0.428039934	0.992100981	0.737482484	P137	P	0.719374379	0.308188957	0.158247548
P63	P	0.37450999	0.167046965	0.640815031	P138	P	0.110125193	0.590406462	0.290221536
P64	P	0.289290325	0.092447548	0.757370998	P139	P	0.451614945	0.430432728	0.565440444
P65	P	0.583193201	0.24050875	0.126498494	P140	P	0.749552812	0.272734858	0.659099273
P66	P	0.242816013	0.964073847	0.455088954	P141	P	0.683064199	0.710270743	0.583931281
P67	P	0.741533407	0.88421046	0.207899913	P142	P	0.237145169	0.368480021	0.545499238
P68	P	0.856658998	0.444954661	0.899611071	P143	P	0.112198883	0.45185598	0.895318415
P69	P	0.410742681	0.130554325	0.192581195	P144	P	0.896694997	0.110402748	0.690427497
P70	P	0.09512558	0.87429773	0.672054396	P145	P	0.712725244	0.779161225	0.078137198
P71	P	0.471094094	0.81252918	0.348522146	P146	P	0.10815314	0.284369532	0.963730187
P72	P	0.362555464	0.706238004	0.325279265	P147	P	0.712798369	0.04925697	0.917801713
P73	P	0.599453335	0.566437226	0.201009211	P148	P	0.758620742	0.520617433	0.779745512
P74	P	0.236495182	0.671469581	0.232667378	P149	P	0.661036332	0.291616813	0.530905137
P75	P	0.065767994	0.459275487	0.222441749	P150	P	0.973404493	0.866674444	0.168862013
P76	P	0.526489846	0.392213814	0.428878401	P151	P	0.018650037	0.643700561	0.439449335
P77	P	0.816360498	0.742924925	0.666225887	P152	P	0.964381193	0.205691279	0.017594404
P78	P	0.533851959	0.403045124	0.155946452	P153	P	0.949364273	0.576485521	0.278525303
P79	P	0.148386916	0.122535905	0.98893655	P154	P	0.307936727	0.273616488	0.771160501
P80	P	0.538672916	0.376702439	0.994225795	P155	P	0.451370714	0.859489134	0.526138871
P81	P	0.679543972	0.252833553	0.965989267	P156	P	0.576703812	0.864964157	0.199374504
P82	P	0.320223452	0.93288911	0.597146177	P157	P	0.403509083	0.786686	0.196803021
P83	P	0.941491436	0.245488725	0.564856587	P158	P	0.181642544	0.471499435	0.372412226
P84	P	0.300524018	0.851784389	0.10028991	P159	P	0.465787011	0.768607728	0.83527749
P85	P	0.185781361	0.102067575	0.136154019	P160	P	0.265735623	0.437453865	0.870562364
P86	P	0.412468823	0.778956444	0.652894596	P161	P	0.810987682	0.587883118	0.978157905
P87	P	0.984564499	0.066089791	0.939032097	P162	P	0.769046145	0.915117176	0.612066129
P88	P	0.743311841	0.431551255	0.648722582	P163	P	0.132247353	0.588156457	0.098019006
P89	P	0.974602721	0.303767771	0.874848211	P164	P	0.508364326	0.923819872	0.95648605
P90	P	0.957249886	0.87461525	0.761489994	P165	P	0.594694294	0.539261679	0.039395682
P91	P	0.901445893	0.207685152	0.17497676	P166	P	0.680590093	0.027450746	0.276711582
P92	P	0.160168259	0.608331541	0.849639423	P167	P	0.754551886	0.129239295	0.596805095
P93	P	0.673454143	0.573659955	0.653911718	P168	P	0.314289947	0.644292495	0.086533923
P94	P	0.375582932	0.944130259	0.359071894	P169	P	0.448668219	0.250354759	0.516826311
P95	P	0.430880484	0.387856578	0.286158887	P170	P	0.794073092	0.365515802	0.504818951
P96	P	0.664764327	0.495993782	0.904459114	P171	P	0.836742455	0.471755973	0.307398454
P97	P	0.202484349	0.260700292	0.656551932	P172	P	0.993045618	0.969513689	0.414606669
P98	P	0.150039992	0.768553658	0.128555102	P173	P	0.685946182	0.953414709	0.041543076
P99	P	0.722554464	0.671273578	0.774694518	P174	P	0.611944684	0.006225254	0.526697403
P100	P	0.968052484	0.60016442	0.605259794	P175	P	0.387608061	0.03919169	0.907575972
P101	P	0.14861214	0.94251721	0.176178932	P176	P	0.386726772	0.27763485	0.11597102
P102	P	0.459621732	0.930698907	0.10585978	P177	P	0.185247639	0.855865695	0.998362494
P103	P	0.29816642	0.864760138	0.783341303	P178	P	0.154840996	0.026605293	0.320999723
P104	P	0.083923666	0.71275666	0.993756136	P179	P	0.903835179	0.879882911	0.322905007
P105	P	0.717280681	0.468351124	0.205938724	P180	P	0.082788753	0.333329722	0.55297723
P106	P	0.290176354	0.713417391	0.733144686	O1	O	0.343185197	0.299016149	0.740362079
P107	P	0.735826366	0.514812682	0.427796222	O2	O	0.809207259	0.469082928	0.922522323
P108	P	0.896321599	0.434437113	0.612442798	O3	O	0.244287738	0.392779783	0.891495621
P109	P	0.993353884	0.531185697	0.049579719	O4	O	0.133594371	0.003562767	0.370744416
P110	P	0.873097074	0.249589306	0.76243675	O5	O	0.121024519	0.812739247	0.876796033
P111	P	0.312815629	0.825258267	0.43782814	O6	O	0.664038752	0.99859247	0.550837531
P112	P	0.075092808	0.987389942	0.570830074	O7	O	0.253905929	0.291051611	0.753809768
P113	P	0.482839732	0.061989718	0.02694182	O8	O	0.354755608	0.012206544	0.221099337
P114	P	0.499332715	0.218049521	0.994398584	O9	O	0.730834352	0.052916578	0.285378075
P115	P	0.260495557	0.350791722	0.015952929	O10	O	0.507922448	0.270453524	0.02577301
P116	P	0.160510323	0.338765938	0.249936093	O11	O	0.472962905	0.382452802	0.429447669
P117	P	0.072220376	0.183170225	0.635176217	O12	O	0.705544173	0.222720262	0.926376893
P118	P	0.430699211	0.574924873	0.160409083	O13	O	0.901992274	0.548174254	0.788953854
P119	P	0.57186856	0.088450913	0.161050197	O14	O	0.54804586	0.385813936	0.206919578
P120	P	0.243689597	0.718967584	0.933974333	O15	O	0.266026523	0.391252593	0.980744239
P121	P	0.029069827	0.018478416	0.760856781	O16	O	0.307283984	0.313983317	0.005797591
P122	P	0.116300241	0.425573705	0.047943988	O17	O	0.711200916	0.143162028	0.380546211
P123	P	0.579747964	0.121917099	0.854309304	O18	O	0.174514277	0.358027695	0.744681213
P124	P	0.016651465	0.583586618	0.906436474	O19	O	0.845214269	0.741464162	0.716835658
P125	P	0.714042785	0.422486532	0.044538906	O20	O	0.904104922	0.003758914	0.104485848
P126	P	0.747255665	0.959150618	0.467044842	O21	O	0.02779626	0.838620315	0.175236915
P127	P	0.532409955	0.727913913	0.137432884	O22	O	0.069722137	0.665924077	0.54448215
P128	P	0.056000531	0.853535627	0.497180191	O23	O	0.433888256	0.494789965	0.992329677
P129	P	0.836409796	0.224356131	0.448169251	O24	O	0.418750396	0.726875997	0.199248192
P130	P	0.073912125	0.13660453	0.450249254	O25	O	0.51371272	0.087599381	0.410506557
P131	P	0.841666608	0.888500203	0.076095614	O26	O	0.018328919	0.653561067	0.142445069
P132	P	0.437448527	0.013739803	0.53477621	O27	O	0.802957139	0.435628008	0.337704483
P133	P	0.829381307	0.004343769	0.245135627	O28	O	0.025007465	0.048678914	0.982577862
P134	P	0.851526392	0.156588904	0.885794704	O29	O	0.316290313	0.817297278	0.378864645
P135	P	0.71280314	0.181523924	0.785556507	O30	O	0.027765607	0.58948436	0.46744468

O31	O	0.257388683	0.517828287	0.507857782	O106	O	0.042329498	0.721486239	0.204234884
O32	O	0.08644402	0.556957824	0.334905638	O107	O	0.316057811	0.217438902	0.766404535
O33	O	0.174561637	0.196193879	0.781667367	O108	O	0.729594614	0.036822056	0.867173882
O34	O	0.186229506	0.085338315	0.000718255	O109	O	0.028369789	0.92383594	0.400683418
O35	O	0.361784012	0.222696351	0.631233022	O110	O	0.560844956	0.597310331	0.175840505
O36	O	0.244347633	0.590369513	0.347777619	O111	O	0.773775114	0.081871634	0.417099159
O37	O	0.701955121	0.662522813	0.55826702	O112	O	0.556832548	0.145207845	0.8125314
O38	O	0.164665787	0.510323635	0.409760358	O113	O	0.414521979	0.327950943	0.963180268
O39	O	0.730228206	0.298856156	0.831138988	O114	O	0.470871764	0.589894987	0.628063702
O40	O	0.569152252	0.49645915	0.336466849	O115	O	0.2065162	0.262894794	0.075517407
O41	O	0.656791784	0.524591677	0.326855662	O116	O	0.464698998	0.296793026	0.540602131
O42	O	0.903913113	0.181578063	0.903932674	O117	O	0.436253701	0.819271832	0.231173696
O43	O	0.4769239	0.204238542	0.830578689	O118	O	0.122987012	0.901309668	0.631723417
O44	O	0.029766042	0.279862824	0.086895675	O119	O	0.053796299	0.759630063	0.009851377
O45	O	0.595037292	0.593835914	0.781860635	O120	O	0.945390337	0.263619993	0.018824277
O46	O	0.6264674	0.230258808	0.973118513	O121	O	0.846974735	0.54164253	0.993658906
O47	O	0.890874018	0.430245214	0.943819774	O122	O	0.361978318	0.153605807	0.497824148
O48	O	0.06562858	0.312263235	0.982331783	O123	O	0.344360534	0.678548249	0.485771344
O49	O	0.379441437	0.306115426	0.420964473	O124	O	0.888818405	0.780600559	0.98986011
O50	O	0.634561034	0.926396432	0.789777329	O125	O	0.574987412	0.332599505	0.974792608
O51	O	0.12140336	0.575901218	0.821379491	O126	O	0.520050333	0.287985146	0.65610239
O52	O	0.64276052	0.991708835	0.023892591	O127	O	0.075292303	0.167289232	0.766858879
O53	O	0.718389408	0.974674077	0.088534914	O128	O	0.711339289	0.838285365	0.201320769
O54	O	0.44061609	0.024452047	0.025482684	O129	O	0.068006917	0.821510588	0.543471558
O55	O	0.277142393	0.762782881	0.920102837	O130	O	0.445359301	0.181685796	0.19324778
O56	O	0.282591452	0.075850429	0.244179246	O131	O	0.717190993	0.827014353	0.888968895
O57	O	0.325284875	0.329367229	0.658219957	O132	O	0.643044724	0.974102964	0.686717378
O58	O	0.15285139	0.473241539	0.323176758	O133	O	0.903646505	0.670626917	0.887776597
O59	O	0.13486298	0.722104954	0.508937895	O134	O	0.994345922	0.197224473	0.970635235
O60	O	0.461681833	0.918366972	0.983117825	O135	O	0.059426243	0.900698756	0.976991422
O61	O	0.496652567	0.69251958	0.117037129	O136	O	0.568447909	0.283290017	0.090644685
O62	O	0.723813515	0.596506045	0.656222588	O137	O	0.970378937	0.626229693	0.248457463
O63	O	0.522091454	0.979574968	0.95753221	O138	O	0.887451674	0.907292201	0.039676474
O64	O	0.344998564	0.908113471	0.551140657	O139	O	0.153178478	0.254594905	0.437861597
O65	O	0.478024354	0.242942558	0.468847518	O140	O	0.155149596	0.419190222	0.917133454
O66	O	0.873985669	0.167983504	0.197819099	O141	O	0.773536507	0.528723969	0.563518227
O67	O	0.335519144	0.662360415	0.307594365	O142	O	0.948445956	0.924437237	0.579782967
O68	O	0.673936657	0.054340884	0.715094129	O143	O	0.841063177	0.401170012	0.869844449
O69	O	0.817919318	0.632362633	0.016393976	O144	O	0.163052132	0.572505754	0.275651856
O70	O	0.695633559	0.232292001	0.763715523	O145	O	0.560443607	0.296134306	0.882748347
O71	O	0.320933322	0.707504979	0.571128676	O146	O	0.815865025	0.018827842	0.11393951
O72	O	0.340972888	0.755202531	0.303744779	O147	O	0.481103839	0.005220877	0.500566942
O73	O	0.660665011	0.910531053	0.060953698	O148	O	0.427334475	0.07023492	0.537203888
O74	O	0.403069977	0.645462502	0.896250119	O149	O	0.674910785	0.304555639	0.947438971
O75	O	0.677782181	0.353154516	0.149259797	O150	O	0.877523045	0.906539383	0.3695228
O76	O	0.873945521	0.083218489	0.13647782	O151	O	0.794509244	0.887143815	0.05081134
O77	O	0.703517433	0.367769109	0.34680332	O152	O	0.895443482	0.710125565	0.803411839
O78	O	0.742017252	0.468422842	0.035351315	O153	O	0.445700013	0.755788657	0.61121044
O79	O	0.353233341	0.14427941	0.176434525	O154	O	0.932982645	0.155467119	0.695673694
O80	O	0.507478388	0.78776371	0.386529018	O155	O	0.93266319	0.76073056	0.881566751
O81	O	0.406095054	0.802115325	0.141365918	O156	O	0.299510826	0.589287	0.090817376
O82	O	0.695708819	0.820896607	0.038495118	O157	O	0.135108051	0.689068069	0.602431461
O83	O	0.668490136	0.698593403	0.448610122	O158	O	0.577465932	0.426999949	0.130882149
O84	O	0.380600649	0.891580575	0.885433205	O159	O	0.736861554	0.484580171	0.375634342
O85	O	0.040597606	0.047619428	0.7082302	O160	O	0.876444007	0.322609207	0.958832091
O86	O	0.13858582	0.362642843	0.293910202	O161	O	0.272536493	0.675981283	0.101210928
O87	O	0.294273902	0.042536754	0.72527584	O162	O	0.389201131	0.894856465	0.34195687
O88	O	0.15210976	0.661701716	0.830167009	O163	O	0.724741529	0.183655828	0.845593008
O89	O	0.063567724	0.410244127	0.185548117	O164	O	0.890547427	0.095465075	0.630697664
O90	O	0.965979705	0.807446199	0.039864507	O165	O	0.316478073	0.287585492	0.827388602
O91	O	0.877985959	0.984443988	0.231196106	O166	O	0.145864328	0.064447711	0.123310542
O92	O	0.094146889	0.561162182	0.123881578	O167	O	0.111148406	0.458843953	0.589245713
O93	O	0.672024156	0.982737818	0.31775263	O168	O	0.641556174	0.52211219	0.017210288
O94	O	0.951362671	0.505619281	0.018299918	O169	O	0.78057418	0.273075795	0.61624581
O95	O	0.51586566	0.74589796	0.546375863	O170	O	0.342553686	0.114431605	0.758202489
O96	O	0.8531717	0.544547964	0.612957184	O171	O	0.027162085	0.102702029	0.452398307
O97	O	0.566422293	0.283571022	0.787339724	O172	O	0.976812019	0.015281801	0.770673172
O98	O	0.12445202	0.433979785	0.100815063	O173	O	0.755847048	0.040760092	0.955969769
O99	O	0.17189153	0.544243846	0.961938023	O174	O	0.547457153	0.118990935	0.198967379
O100	O	0.457773491	0.818503396	0.810480346	O175	O	0.95831908	0.881185672	0.326757883
O101	O	0.422549278	0.813239835	0.371148191	O176	O	0.404148333	0.994365831	0.933817753
O102	O	0.929583039	0.831960476	0.175837035	O177	O	0.039584493	0.515784099	0.028072366
O103	O	0.223406218	0.681767812	0.287218237	O178	O	0.342424155	0.548531973	0.500083035
O104	O	0.744108387	0.803786719	0.116994905	O179	O	0.212616318	0.836381138	0.6333891
O105	O	0.14914927	0.383436125	0.027745407	O180	O	0.110686122	0.792388726	0.096963182

O181	O	0.401706506	0.164119676	0.692886564	O256	O	0.076222357	0.58335519	0.242382755
O182	O	0.894300999	0.470534539	0.329973588	O257	O	0.305778188	0.41194618	0.704008235
O183	O	0.877820384	0.22083572	0.123321483	O258	O	0.2533867	0.546025516	0.924784821
O184	O	0.201651778	0.319515461	0.540951349	O259	O	0.750318885	0.449824506	0.161982879
O185	O	0.785549091	0.139292999	0.217522818	O260	O	0.35463288	0.0462628	0.092880767
O186	O	0.139513884	0.285444221	0.244641535	O261	O	0.051242889	0.275843801	0.181528616
O187	O	0.194363184	0.946838257	0.141889394	O262	O	0.693571671	0.551235891	0.431147935
O188	O	0.548238396	0.370252638	0.827306329	O263	O	0.08797137	0.005194056	0.622363364
O189	O	0.015038629	0.189232075	0.631607975	O264	O	0.990535898	0.131713487	0.793530957
O190	O	0.86431206	0.021645058	0.499953393	O265	O	0.126803525	0.743322135	0.170695001
O191	O	0.475156668	0.34461898	0.719380741	O266	O	0.549661858	0.525416211	0.999507525
O192	O	0.167785606	0.13230915	0.853760166	O267	O	0.755831311	0.085365294	0.567222327
O193	O	0.496258675	0.012299554	0.316303116	O268	O	0.988718504	0.589244032	0.045604984
O194	O	0.767754077	0.329313838	0.297603919	O269	O	0.410557686	0.106768547	0.239989185
O195	O	0.226057937	0.473001528	0.848666589	O270	O	0.718938346	0.269588101	0.347480891
O196	O	0.394062137	0.641638836	0.803891939	O271	O	0.438169248	0.603867653	0.711450478
O197	O	0.331363499	0.602987925	0.861774998	O272	O	0.255322594	0.721977669	0.783564323
O198	O	0.141036593	0.222904744	0.860348064	O273	O	0.212503796	0.316946159	0.656243488
O199	O	0.77876925	0.158400376	0.305324686	O274	O	0.77163901	0.699590512	0.790852309
O200	O	0.608489939	0.499556171	0.924146252	O275	O	0.229803089	0.31314424	0.156573001
O201	O	0.63490251	0.543498478	0.156861143	O276	O	0.34394016	0.839561862	0.759367848
O202	O	0.260448181	0.817662281	0.4496852	O277	O	0.576263922	0.677204381	0.247731355
O203	O	0.762903152	0.361540243	0.456108055	O278	O	0.32941809	0.460936952	0.5339921
O204	O	0.06122247	0.406435827	0.042786559	O279	O	0.293296416	0.174925517	0.554354365
O205	O	0.127282315	0.580590687	0.036940874	O280	O	0.789503885	0.978043779	0.213574022
O206	O	0.478572808	0.409692541	0.308687634	O281	O	0.103855486	0.915305474	0.150119467
O207	O	0.507542819	0.924782284	0.077140088	O282	O	0.174279183	0.849100197	0.542928964
O208	O	0.718007844	0.752098055	0.570220459	O283	O	0.030474585	0.299801077	0.862927577
O209	O	0.94127969	0.853733927	0.706778817	O284	O	0.813682573	0.036607637	0.785115011
O210	O	0.75131221	0.663645443	0.455121716	O285	O	0.969313038	0.353707176	0.911002869
O211	O	0.724593353	0.282109388	0.111832301	O286	O	0.646968512	0.733089701	0.251921302
O212	O	0.784394116	0.554287015	0.820466744	O287	O	0.702425625	0.614677775	0.155254723
O213	O	0.931268514	0.067362711	0.965897142	O288	O	0.57784303	0.658021507	0.717878383
O214	O	0.485240982	0.932557057	0.263520554	O289	O	0.321853078	0.852372251	0.04614811
O215	O	0.78157	0.177187666	0.568598712	O290	O	0.694097449	0.105893384	0.921830046
O216	O	0.551364585	0.374350979	0.3839212	O291	O	0.592197418	0.969783426	0.864762851
O217	O	0.133391244	0.996252456	0.191120424	O292	O	0.719506642	0.911957599	0.252935987
O218	O	0.467577286	0.544951408	0.186547671	O293	O	0.381019398	0.084814467	0.939079573
O219	O	0.099492527	0.365867786	0.803007992	O294	O	0.958834982	0.590425229	0.926141617
O220	O	0.356545585	0.831890988	0.951765636	O295	O	0.241474315	0.496192268	0.002007619
O221	O	0.578163916	0.90607885	0.159080725	O296	O	0.812952545	0.878390773	0.601187826
O222	O	0.542435998	0.76990031	0.093452828	O297	O	0.075829899	0.544824351	0.566397536
O223	O	0.986171152	0.021069364	0.89678513	O298	O	0.552463649	0.037684014	0.158432112
O224	O	0.366245863	0.536867719	0.05093366	O299	O	0.589080761	0.507524515	0.089104056
O225	O	0.322646743	0.952948283	0.358274225	O300	O	0.424556897	0.707955914	0.065282871
O226	O	0.60757336	0.69864993	0.330656347	O301	O	0.612884523	0.996853195	0.466255084
O227	O	0.760373911	0.567920794	0.977297573	O302	O	0.210219111	0.590375829	0.838236134
O228	O	0.474482522	0.507561976	0.879271192	O303	O	0.264498555	0.374554312	0.071682406
O229	O	0.896837642	0.227117941	0.447118786	O304	O	0.252723145	0.232303494	0.655104404
O230	O	0.457994999	0.895118423	0.146234276	O305	O	0.126156157	0.878879428	0.812516519
O231	O	0.33733682	0.695133534	0.856847072	O306	O	0.563887761	0.111953241	0.110144662
O232	O	0.716124527	0.942502385	0.998674802	O307	O	0.482368115	0.018949583	0.224647734
O233	O	0.25693612	0.865060901	0.743405277	O308	O	0.958799451	0.252950963	0.904033914
O234	O	0.684111311	0.703050307	0.639387912	O309	O	0.824745093	0.602455127	0.921967645
O235	O	0.703740342	0.436563163	0.611413442	O310	O	0.974669902	0.768844418	0.372165812
O236	O	0.852363609	0.83529455	0.094945959	O311	O	0.529011431	0.036239138	0.031274944
O237	O	0.345621985	0.91085278	0.648055843	O312	O	0.50682152	0.395189386	0.953722751
O238	O	0.575936965	0.676457873	0.814437524	O313	O	0.99879711	0.119160773	0.91275292
O239	O	0.840658424	0.736438175	0.882149587	O314	O	0.217321885	0.336478643	0.258815044
O240	O	0.879657407	0.556596944	0.70249819	O315	O	0.506090791	0.620473831	0.772771548
O241	O	0.408147256	0.14384955	0.598603715	O316	O	0.52629001	0.852961108	0.211716169
O242	O	0.661011523	0.864415225	0.954569176	O317	O	0.835211293	0.457808181	0.25128769
O243	O	0.528744837	0.658474103	0.579380157	O318	O	0.766314694	0.765254354	0.67493705
O244	O	0.395083059	0.646805107	0.645030474	O319	O	0.016445959	0.62008719	0.619565179
O245	O	0.463330802	0.031439628	0.744702547	O320	O	0.932049862	0.310303833	0.381131536
O246	O	0.095533688	0.719712837	0.938255733	O321	O	0.425273729	0.89877221	0.500278022
O247	O	0.136731741	0.317185594	0.927021144	O322	O	0.503464377	0.359810271	0.037389512
O248	O	0.503392515	0.909762068	0.903766382	O323	O	0.70942284	0.915876239	0.478543708
O249	O	0.624058453	0.049649519	0.638067218	O324	O	0.871127741	0.463808759	0.655334926
O250	O	0.426231058	0.164163075	0.902355997	O325	O	0.36268737	0.277346636	0.164399626
O251	O	0.141227141	0.267697273	0.012329362	O326	O	0.008076822	0.877101633	0.503302249
O252	O	0.433746407	0.447199199	0.81886157	O327	O	0.426841422	0.294951692	0.654530469
O253	O	0.129140836	0.643713866	0.114140746	O328	O	0.276516554	0.230405065	0.139360722
O254	O	0.932034586	0.841943594	0.803500461	O329	O	0.019837875	0.012022575	0.435180311
O255	O	0.37658929	0.01093535	0.709220023	O330	O	0.880568398	0.482903433	0.869898889

O331	O	0.4183205	0.699088943	0.31336471
O332	O	0.148720981	0.606092856	0.901689974
O333	O	0.171320571	0.421661922	0.397623438
O334	O	0.629651244	0.602807838	0.233035878
O335	O	0.039596742	0.876761068	0.658078856
O336	O	0.688978664	0.375568672	0.793073998
O337	O	0.374316226	0.554747375	0.174989581
O338	O	0.682269816	0.316952829	0.274612925
O339	O	0.438043825	0.633248351	0.176384581
O340	O	0.119886777	0.471197029	0.018922606
O341	O	0.36152799	0.361141953	0.494238034
O342	O	0.674877179	0.068207306	0.415300577
O343	O	0.299124348	0.962396106	0.46823351
O344	O	0.166093031	0.893676805	0.957271939
O345	O	0.726249164	0.898861063	0.582292092
O346	O	0.280546097	0.834182762	0.832169149
O347	O	0.261010453	0.81321626	0.257306958
O348	O	0.260226746	0.59661556	0.439893603
O349	O	0.699556022	0.144099405	0.606376829
O350	O	0.165518397	0.797592009	0.798775088
O351	O	0.346030103	0.517151749	0.96529199
O352	O	0.927719516	0.533890903	0.240195459
O353	O	0.175510927	0.331735324	0.837113682
O354	O	0.264108207	0.891173464	0.213434721
O355	O	0.605929202	0.885909524	0.245145479
O356	O	0.288402779	0.355163903	0.548843258
O357	O	0.424130435	0.391772356	0.901142808
O358	O	0.114065193	0.257827924	0.356826119
O359	O	0.229172167	0.405071197	0.503389231
O360	O	0.803102989	0.939579449	0.483758394
O361	O	0.698215424	0.272949785	0.198375661
O362	O	0.240055454	0.67480703	0.387295291
O363	O	0.627108345	0.086225744	0.172523666
O364	O	0.223965484	0.397760061	0.597400897
O365	O	0.176945309	0.24494555	0.704319758
O366	O	0.940123903	0.930855952	0.767728444
O367	O	0.843535757	0.579880461	0.529213864
O368	O	0.826550814	0.058599708	0.240180339
O369	O	0.107557313	0.368693006	0.520652637
O370	O	0.920302839	0.17442215	0.020682985
O371	O	0.443511532	0.271185228	0.126605786
O372	O	0.892150952	0.900905281	0.648924594
O373	O	0.262588285	0.720410739	0.205942429
O374	O	0.784047466	0.799769326	0.791919758
O375	O	0.018520227	0.539302563	0.864161663
O376	O	0.992259164	0.511331608	0.105016855
O377	O	0.402319852	0.984623919	0.332525357
O378	O	0.675342767	0.51947984	0.676513988
O379	O	0.159591904	0.86846437	0.044443356
O380	O	0.113599237	0.0340075	0.286682968
O381	O	0.994962299	0.552592156	0.312663796
O382	O	0.421306049	0.754543554	0.867711215
O383	O	0.3854065	0.407460731	0.308740489
O384	O	0.908206427	0.592043239	0.310183155
O385	O	0.923274739	0.6390676	0.618328274
O386	O	0.165608986	0.91778367	0.221490679
O387	O	0.971079445	0.311008704	0.296571895
O388	O	0.66581281	0.46322248	0.188849404
O389	O	0.104537144	0.89750219	0.723469205
O390	O	0.411835364	0.816684902	0.540081259
O391	O	0.785811219	0.399136597	0.629747776
O392	O	0.452432992	0.983978269	0.124973356
O393	O	0.637122425	0.064073481	0.284810211
O394	O	0.230517608	0.209548055	0.853040463
O395	O	0.997200349	0.194174311	0.064480685
O396	O	0.239629478	0.863922434	0.004035384
O397	O	0.778765284	0.031253538	0.700815101
O398	O	0.963418254	0.988811248	0.369434029
O399	O	0.814057437	0.882431259	0.81277561
O400	O	0.458373362	0.201939769	0.552885669
O401	O	0.99650013	0.215617848	0.762593131
O402	O	0.55914655	0.762306093	0.27624588
O403	O	0.424678485	0.537885936	0.807377827
O404	O	0.112876136	0.334977873	0.414068657
O405	O	0.736853752	0.636213424	0.736722223
O406	O	0.738719557	0.914427849	0.15916284
O407	O	0.609521583	0.012646143	0.788229083
O408	O	0.699677109	0.299619236	0.649981859
O409	O	0.421799137	0.837857386	0.660514275
O410	O	0.684773003	0.921274168	0.880472168
O411	O	0.900765659	0.254818437	0.214252747
O412	O	0.723527801	0.41509906	0.696677483
O413	O	0.094803921	0.198529561	0.581114161
O414	O	0.362161081	0.65763907	0.116250979
O415	O	0.432595373	0.39383954	0.228836772
O416	O	0.946061414	0.611158538	0.733086285
O417	O	0.356258213	0.772768476	0.643152914
O418	O	0.706180716	0.249392943	0.012771524
O419	O	0.114756932	0.483289372	0.21413707
O420	O	0.486872954	0.305214473	0.827002136
O421	O	0.826964547	0.24693021	0.978992166
O422	O	0.928174515	0.707120957	0.326565363
O423	O	0.667712114	0.758461978	0.098881772
O424	O	0.53307922	0.450098141	0.431040941
O425	O	0.266561791	0.82162933	0.555904581
O426	O	0.702952911	0.500431985	0.950009392
O427	O	0.218644761	0.927102231	0.486692778
O428	O	0.387712829	0.697917728	0.982790795
O429	O	0.77319976	0.299584271	0.702368153
O430	O	0.816668025	0.22941903	0.504358407
O431	O	0.958532486	0.721248464	0.000533236
O432	O	0.593346289	0.063789384	0.533419661
O433	O	0.445173904	0.9912112	0.58682382
O434	O	0.818765771	0.269848837	0.417877145
O435	O	0.334027053	0.988780555	0.594975498
O436	O	0.108591683	0.820796495	0.670642251
O437	O	0.294276882	0.466615692	0.91093778
O438	O	0.671513482	0.435229004	0.082282601
O439	O	0.950992141	0.947936475	0.454228073
O440	O	0.758027773	0.163805599	0.754599407
O441	O	0.737128316	0.737431177	0.399549218
O442	O	0.237653951	0.077457331	0.127901835
O443	O	0.992456061	0.679829998	0.475683575
O444	O	0.635089722	0.448652231	0.384314847
O445	O	0.219603979	0.01475331	0.462413931
O446	O	0.579056382	0.528345438	0.232820617
O447	O	0.182716012	0.114311954	0.189352345
O448	O	0.433670847	0.092289836	0.153754162
O449	O	0.617584826	0.085767751	0.840052876
O450	O	0.022225316	0.468437826	0.553147556
O451	O	0.174703895	0.613901057	0.40046184
O452	O	0.622431094	0.846999159	0.871529112
O453	O	0.335074396	0.875752855	0.273419914
O454	O	0.446933359	0.202514023	0.99467605
O455	O	0.550214693	0.888678936	0.98398211
O456	O	0.338669975	0.415411148	0.620241961
O457	O	0.280042471	0.131578636	0.967780445
O458	O	0.251408177	0.126577704	0.732101985
O459	O	0.214646823	0.138798661	0.644596611
O460	O	0.189477476	0.658651418	0.206318896
O461	O	0.064993647	0.420032862	0.896348057
O462	O	0.652146358	0.330098261	0.864851414
O463	O	0.846437645	0.387401916	0.490186
O464	O	0.916683103	0.065817408	0.719720504
O465	O	0.704611019	0.540154055	0.770715543
O466	O	0.344572525	0.350064918	0.908239818
O467	O	0.065236875	0.028142778	0.537429699
O468	O	0.010873433	0.870607127	0.767708871
O469	O	0.228453446	0.725827528	0.992392327
O470	O	0.32822407	0.879624269	0.451073818
O471	O	0.294319031	0.886875051	0.913977251
O472	O	0.952744996	0.055487269	0.522558712
O473	O	0.725932906	0.376682076	0.884224427
O474	O	0.625460207	0.719958642	0.566253001
O475	O	0.24837449	0.889905206	0.303172572
O476	O	0.094526919	0.89832137	0.489320246
O477	O	0.400891938	0.589255308	0.978288391
O478	O	0.189607382	0.233120525	0.165820889
O479	O	0.729865683	0.871422977	0.767412414
O480	O	0.357945483	0.922688655	0.967773397

O481	O	0.860066203	0.060585235	0.047953895	O556	O	0.392783652	0.990253928	0.51399982
O482	O	0.299099401	0.344579349	0.430354431	O557	O	0.129653491	0.111329376	0.934676282
O483	O	0.340768531	0.868548802	0.132453465	O558	O	0.078114099	0.951916686	0.048612436
O484	O	0.993260532	0.232077234	0.55453733	O559	O	0.109671106	0.642367374	0.306812164
O485	O	0.763114308	0.963708088	0.76558829	O560	O	0.126726869	0.960825308	0.550008791
O486	O	0.106009654	0.118450886	0.028017744	O561	O	0.970998937	0.586587662	0.549832062
O487	O	0.941584034	0.276696843	0.612427534	O562	O	0.767730846	0.400537577	0.539817862
O488	O	0.54880713	0.36799818	0.474492355	O563	O	0.441903336	0.568465627	0.103012567
O489	O	0.694335964	0.402435456	0.997974179	O564	O	0.671589382	0.019701339	0.933509232
O490	O	0.322402222	0.655746973	0.028203314	O565	O	0.19552556	0.307351607	0.371354879
O491	O	0.152800472	0.370376281	0.203018019	O566	O	0.381089744	0.330777941	0.09022564
O492	O	0.729014951	0.52325103	0.217455388	O567	O	0.597300423	0.261147156	0.174259453
O493	O	0.233966977	0.949361212	0.400801322	O568	O	0.508316632	0.408113877	0.559944653
O494	O	0.921069888	0.274422628	0.732807244	O569	O	0.685813558	0.655065324	0.068555233
O495	O	0.418459516	0.963422266	0.784323905	O570	O	0.478325485	0.677131508	0.995189904
O496	O	0.958336419	0.187982143	0.169776202	O571	O	0.086354587	0.208244594	0.685576159
O497	O	0.515112499	0.752031539	0.182053038	O572	O	0.065977953	0.184031499	0.476045874
O498	O	0.659198216	0.572627229	0.59381979	O573	O	0.343269432	0.718667892	0.748381086
O499	O	0.75813	0.919616794	0.664478218	O574	O	0.280477541	0.756262016	0.694465872
O500	O	0.943239289	0.308863213	0.830428407	O575	O	0.676902906	0.009128852	0.226525148
O501	O	0.470708368	0.254510373	0.725237111	O576	O	0.887296808	0.906979834	0.278510534
O502	O	0.894354494	0.217481224	0.803524339	O577	O	0.783023377	0.325316858	0.974162862
O503	O	0.275278282	0.63379894	0.533333643	O578	O	0.271023964	0.630136649	0.230013254
O504	O	0.85250561	0.622353293	0.756757303	O579	O	0.950683211	0.433563605	0.624193112
O505	O	0.764572613	0.48478791	0.658546043	O580	O	0.773754188	0.631891691	0.094521251
O506	O	0.565399839	0.986402369	0.25490525	O581	O	0.482419995	0.67198248	0.67362856
O507	O	0.099739449	0.222148851	0.12165235	O582	O	0.516121384	0.767586902	0.863998366
O508	O	0.128957691	0.279541624	0.774764785	O583	O	0.059012488	0.440953763	0.272624313
O509	O	0.020671506	0.708788415	0.321329155	O584	O	0.488745213	0.437750791	0.155449527
O510	O	0.194268088	0.988840267	0.296702804	O585	O	0.432293755	0.331383678	0.30047546
O511	O	0.580606664	0.699000465	0.148736528	O586	O	0.553363728	0.683156353	0.491519967
O512	O	0.728544918	0.431032603	0.253305075	O587	O	0.338636124	0.027582244	0.880800738
O513	O	0.131543436	0.707208264	0.02304364	O588	O	0.056515207	0.034632471	0.118456251
O514	O	0.067158881	0.661439501	0.424577243	O589	O	0.299683384	0.979494258	0.119748585
O515	O	0.282191209	0.115558	0.483212478	O590	O	0.276307712	0.2227526	0.989389516
O516	O	0.174333198	0.245557013	0.613510196	O591	O	0.960762174	0.551493673	0.634372417
O517	O	0.473821049	0.875813777	0.572387166	O592	O	0.697582984	0.640573526	0.821187397
O518	O	0.845046666	0.776180086	0.633774589	O593	O	0.467127477	0.727946195	0.78866883
O519	O	0.918293973	0.279925835	0.525067683	O594	O	0.595512132	0.598073621	0.055484037
O520	O	0.462793481	0.679445237	0.512515375	O595	O	0.269987158	0.07534945	0.811930591
O521	O	0.311071715	0.91763171	0.793871609	O596	O	0.178322925	0.090641357	0.578183365
O522	O	0.05103945	0.6694703	0.999233224	O597	O	0.736256593	0.01062961	0.499565949
O523	O	0.100405686	0.486555293	0.495145092	O598	O	0.702507188	0.646340632	0.38118951
O524	O	0.710786721	0.012608302	0.642990553	O599	O	0.085648675	0.282818209	0.535428041
O525	O	0.154902453	0.632107082	0.530981318	O600	O	0.05508099	0.821571751	0.445146406
O526	O	0.371834479	0.399869514	0.410002987	O601	O	0.023688125	0.195321768	0.848892671
O527	O	0.576329338	0.973044944	0.54945901	O602	O	0.787092923	0.53926586	0.440143674
O528	O	0.491997848	0.835542338	0.486970863	O603	O	0.048714536	0.567261244	0.947445018
O529	O	0.264259056	0.995288804	0.201649807	O604	O	0.426384494	0.053277075	0.865654989
O530	O	0.302307644	0.152408733	0.054868303	O605	O	0.402325039	0.246172564	0.872046137
O531	O	0.801532321	0.315399067	0.530732429	O606	O	0.744730995	0.970769955	0.40881551
O532	O	0.109980687	0.313909892	0.121255539	O607	O	0.086787429	0.142926541	0.398574843
O533	O	0.284844577	0.799185592	0.118353626	O608	O	0.873878679	0.11653065	0.515370056
O534	O	0.059673758	0.047197596	0.804438167	O609	O	0.987970295	0.633242607	0.391777927
O535	O	0.741165098	0.743781044	0.049369314	O610	O	0.749839139	0.383917951	0.069365233
O536	O	0.51713165	0.354714494	0.128173558	O611	O	0.885759703	0.823502865	0.320234296
O537	O	0.880157681	0.323334916	0.301662217	O612	O	0.026648509	0.498451332	0.210276415
O538	O	0.024970721	0.345641399	0.560809095	O613	O	0.123603533	0.472007467	0.845916977
O539	O	0.417674653	0.919117933	0.067890045	O614	O	0.359643562	0.711741246	0.382317777
O540	O	0.674062237	0.34503319	0.557308254	O615	O	0.671073751	0.539887966	0.870339387
O541	O	0.983850784	0.080176883	0.086243796	O616	O	0.46409889	0.13226204	0.470598561
O542	O	0.425369146	0.747573053	0.70331621	O617	O	0.279148751	0.657720807	0.710547204
O543	O	0.297090712	0.211954454	0.462159277	O618	O	0.198229802	0.723015758	0.904874297
O544	O	0.035963429	0.949251029	0.5707751	O619	O	0.436260913	0.463661361	0.52056924
O545	O	0.630910384	0.211024164	0.102167018	O620	O	0.536297183	0.201734129	0.133189829
O546	O	0.913264428	0.071734764	0.432179634	O621	O	0.379822831	0.497292492	0.880721217
O547	O	0.487419476	0.865692079	0.339159953	O622	O	0.606903377	0.160482286	0.88885642
O548	O	0.390524069	0.171748988	0.817659564	O623	O	0.028294827	0.740503683	0.113249481
O549	O	0.251661021	0.888276064	0.106394911	O624	O	0.536340036	0.091051413	0.88300512
O550	O	0.041458274	0.869945686	0.060529807	O625	O	0.239032535	0.587207312	0.005601377
O551	O	0.710830194	0.562633041	0.072976264	O626	O	0.872281169	0.381377458	0.611910087
O552	O	0.462373816	0.156510668	0.379093776	O627	O	0.183694499	0.567307377	0.112419621
O553	O	0.793145059	0.61009908	0.598836375	O628	O	0.273239897	0.02893865	0.043530417
O554	O	0.262663452	0.721956691	0.498434002	O629	O	0.446264383	0.460566953	0.610071782
O555	O	0.47509407	0.101495897	0.067212875	O630	O	0.239729262	0.475465096	0.358483228

O631	O	0.612783576	0.274451588	0.551689998	O706	O	0.823773309	0.134676482	0.926772292
O632	O	0.367808282	0.239918114	0.083064498	O707	O	0.120147902	0.110408204	0.476953873
O633	O	0.111262373	0.335557185	0.606456399	O708	O	0.473950252	0.779841366	0.302489304
O634	O	0.17531024	0.805440332	0.982218795	O709	O	0.982689989	0.052909729	0.172889902
O635	O	0.81477726	0.526390719	0.311680195	O710	O	0.104132481	0.491759834	0.933043892
O636	O	0.090515973	0.238271082	0.935602029	O711	O	0.2149584	0.321535594	0.00843516
O637	O	0.480486542	0.089040656	0.977823161	O712	O	0.034841249	0.633778506	0.887280895
O638	O	0.352573501	0.790989488	0.214203818	O713	O	0.90809107	0.743314325	0.072532214
O639	O	0.81301707	0.693352565	0.647458876	O714	O	0.786052217	0.966351919	0.590804427
O640	O	0.424108351	0.077123817	0.412635295	O715	O	0.88798638	0.456185475	0.563673777
O641	O	0.674105161	0.144124589	0.779163792	O716	O	0.624829767	0.446370533	0.286070638
O642	O	0.186367896	0.808513386	0.144684326	O717	O	0.514618438	0.221887715	0.938267938
O643	O	0.052558525	0.967536829	0.755020308	O718	O	0.245728249	0.055532567	0.62756691
O644	O	0.969989842	0.907157491	0.210333209	O719	O	0.86210291	0.115723544	0.843626383
O645	O	0.393023801	0.255104745	0.506554942	O720	O	0.179550932	0.148679111	0.102499977
O646	O	0.941455329	0.394387709	0.334812825	N1	N	0.507568725	0.559626032	0.406019753
O647	O	0.842339162	0.28974711	0.787316841	N2	N	0.590913476	0.242540885	0.357190997
O648	O	0.444369774	0.768811989	0.001715988	N3	N	0.624478962	0.846444848	0.391651346
O649	O	0.268429977	0.667417334	0.925172032	N4	N	0.850797625	0.892861249	0.928823397
O650	O	0.709813458	0.136220019	0.47070113	N5	N	0.051635687	0.733765062	0.748684567
O651	O	0.74290799	0.216992693	0.674652725	N6	N	0.980747184	0.458699527	0.426522191
O652	O	0.179974617	0.073775261	0.33223396	N7	N	0.87814135	0.754173573	0.50976463
O653	O	0.778203486	0.117956407	0.648514436	N8	N	0.273132517	0.447656686	0.156080355
O654	O	0.328024288	0.140631645	0.641213175	N9	N	0.000836375	0.418132538	0.755390699
O655	O	0.081869323	0.130088327	0.640727464	N10	N	0.58406717	0.466042126	0.705284721
O656	O	0.706472372	0.254565679	0.54590627	N11	N	0.550644614	0.830079065	0.680584002
O657	O	0.455344246	0.952186282	0.69993397	N12	N	0.827197015	0.716051468	0.219735084
O658	O	0.410957259	0.385689724	0.566533209	N13	N	0.612804877	0.664348804	0.938955583
O659	O	0.760010318	0.467479315	0.791720691	N14	N	0.885123986	0.449371399	0.11636578
O660	O	0.043146531	0.123697219	0.136862456	N15	N	0.104704591	0.82019559	0.317564893
O661	O	0.34593875	0.969022697	0.043860408	N16	N	0.180733863	0.995688959	0.864893908
O662	O	0.198089194	0.855408933	0.860841982	N17	N	0.714881989	0.140804765	0.100687412
O663	O	0.657494317	0.302171473	0.470364136	N18	N	0.295105322	0.188950094	0.30599626
O664	O	0.161007761	0.068041693	0.663832267	N19	N	0.570925465	0.149785997	0.635924563
O665	O	0.841217798	0.218828147	0.722256975	N20	N	0.278669502	0.528570825	0.712643988
O666	O	0.954598527	0.970819034	0.668097882	N21	N	0.941149203	0.136147766	0.336293722
O667	O	0.537702635	0.952062777	0.781944161	C1	C	0.543272446	0.242348734	0.332177536
O668	O	0.349673336	0.788212273	0.468284895	C2	C	0.560085133	0.809712437	0.729256366
O669	O	0.359073096	0.174086063	0.984519178	C3	C	0.833646754	0.688326747	0.173548489
O670	O	0.392151084	0.946440236	0.416133123	C4	C	0.816488112	0.932120674	0.915965458
O671	O	0.304276424	0.424563584	0.82950287	C5	C	0.545145643	0.506613146	0.699772581
O672	O	0.911695468	0.194723894	0.570834912	C6	C	0.618821894	0.197265576	0.345594876
O673	O	0.821577441	0.993006181	0.300795764	C7	C	0.007450572	0.713204262	0.72728264
O674	O	0.727383461	0.477728565	0.469220783	C8	C	0.097292638	0.705775499	0.726618557
O675	O	0.973380337	0.888067944	0.116475122	C9	C	0.225740671	0.468775816	0.173700895
O676	O	0.970213367	0.780926846	0.276676493	C10	C	0.950386118	0.486641127	0.462433561
O677	O	0.57152511	0.416602994	0.011706926	C11	C	0.607124102	0.699825207	0.982227269
O678	O	0.815277187	0.851016182	0.723666295	C12	C	0.838489978	0.683493863	0.261386533
O679	O	0.266265433	0.930698581	0.589744043	C13	C	0.756258787	0.175886457	0.105992716
O680	O	0.604624903	0.820430426	0.179644358	C14	C	0.226374133	0.52575543	0.693835569
O681	O	0.288808356	0.997474162	0.288502783	C15	C	0.114958049	0.863789768	0.348312717
O682	O	0.179971601	0.731087638	0.097692735	C16	C	0.014233464	0.453609336	0.714424711
O683	O	0.851680901	0.972386539	0.725051738	C17	C	0.137762781	0.777368827	0.32882141
O684	O	0.372795093	0.411285564	0.974573639	C18	C	0.504234033	0.587919073	0.36132702
O685	O	0.198629341	0.766369697	0.576008898	C19	C	0.008699266	0.366531639	0.742026282
O686	O	0.688572633	0.710627619	0.756312391	C20	C	0.524420473	0.151837737	0.664589892
O687	O	0.844654298	0.123331921	0.708132678	C21	C	0.913644877	0.761940861	0.469854729
O688	O	0.79018367	0.226113198	0.241185474	C22	C	0.967229414	0.088641565	0.319482491
O689	O	0.956643843	0.717338361	0.172457336	C23	C	0.70946516	0.128167919	0.047026311
O690	O	0.708432754	0.175575479	0.250108102	C24	C	0.349359955	0.191272301	0.310920712
O691	O	0.636702392	0.602096763	0.680415526	C25	C	0.304484324	0.436313957	0.199626533
O692	O	0.821258663	0.198247997	0.86174354	C26	C	0.23266346	0.997277681	0.881821919
O693	O	0.834553416	0.923373442	0.126277857	C27	C	0.6679015	0.653381992	0.935884932
O694	O	0.670427054	0.447350392	0.883562222	C28	C	0.002445878	0.41398625	0.448214971
O695	O	0.173059094	0.173496702	0.993650168	C29	C	0.557849143	0.416140063	0.691370395
O696	O	0.794604958	0.866813698	0.216513849	C30	C	0.609004755	0.17442829	0.665212563
O697	O	0.785965354	0.530154408	0.733466096	C31	C	0.148756707	0.994183187	0.912740546
O698	O	0.392836958	0.385482542	0.685259362	C32	C	0.963759702	0.179539666	0.305923846
O699	O	0.76885507	0.334175424	0.174194747	C33	C	0.311736406	0.552587166	0.674425793
O700	O	0.816744072	0.173001351	0.419907643	C34	C	0.458448851	0.54482018	0.426172781
O701	O	0.313638004	0.483621387	0.441061998	C35	C	0.827741473	0.752662923	0.493123854
O702	O	0.815388742	0.282938924	0.895907847	C36	C	0.630234501	0.899887292	0.396517531
O703	O	0.987202628	0.942817043	0.022523229	C37	C	0.659690512	0.830223902	0.348314946
O704	O	0.878401729	0.985516975	0.600184864	C38	C	0.273507315	0.234577978	0.327024383
O705	O	0.530377599	0.173401351	0.017377292	C39	C	0.871040635	0.412883276	0.153786082

C40	C	0.595618748	0.816741067	0.649142708	H10	H	0.76337799	0.418006622	0.167849749
C41	C	0.894420267	0.892300442	0.898351704	H11	H	0.948820613	0.046721791	0.175596987
C42	C	0.893434783	0.425176508	0.068406292	H12	H	0.223673622	0.014380975	0.957199418
C43	C	0.038706662	0.366311994	0.694473985	H13	H	0.233474832	0.615830633	0.849471835
C44	C	0.869415509	0.650144356	0.18121239	H14	H	0.630066706	0.680583891	0.91353561
C45	C	0.11773087	0.746361011	0.372744202	H15	H	0.302437389	0.510402744	0.420398809
C46	C	0.008641715	0.198724043	0.332351273	H16	H	0.719571788	0.833777534	0.025739598
C47	C	0.285152672	0.564305859	0.628327002	H17	H	0.884557603	0.320948037	0.988602532
C48	C	0.607932987	0.464791771	0.757896514	H18	H	0.359462551	0.211338526	0.290555032
C49	C	0.082088469	0.654145337	0.70922738	H19	H	0.80432852	0.676799947	0.165483507
C50	C	0.58750956	0.202873071	0.710464573	H20	H	0.231945449	0.472522516	0.245308751
C51	C	0.544792703	0.559567888	0.447169437	H21	H	0.524524128	0.575168204	0.3412221
C52	C	0.7109483	0.852677873	0.352712786	H22	H	0.88718124	0.872078152	0.870796191
C53	C	0.181157915	0.0059048	0.812292398	H23	H	0.740273918	0.1423687	0.486681592
C54	C	0.36412937	0.209182752	0.362787466	H24	H	0.960627864	0.349804381	0.451101363
C55	C	0.842604717	0.972536068	0.892928061	H25	H	0.535044276	0.177015614	0.579490533
C56	C	0.283384159	0.555883473	0.760447232	H26	H	0.999913132	0.709672665	0.466301183
C57	C	0.693144439	0.69335557	0.961473915	H27	H	0.55327102	0.176396967	0.316634765
C58	C	0.159166019	0.858419072	0.378103016	H28	H	0.768320699	0.86393417	0.544604371
C59	C	0.812497204	0.803974576	0.485833749	H29	H	0.420916798	0.100986015	0.267065136
C60	C	0.939120684	0.873807639	0.928677761	H30	H	0.932148473	0.444725503	0.743390889
C61	C	0.946332297	0.425764204	0.765071145	H31	H	0.997145919	0.159843048	0.072823919
C62	C	0.099271015	0.975055569	0.89776225	H32	H	0.348850681	0.336597246	0.08227847
C63	C	0.582942303	0.245034962	0.411842152	H33	H	0.622219898	0.398158391	0.663005949
C64	C	0.86386168	0.379222196	0.070629636	H34	H	0.870564416	0.766733423	0.24787209
C65	C	0.964208175	0.380063394	0.469569053	H35	H	0.522692302	0.014358451	0.332265498
C66	C	0.58219409	0.621057566	0.914996125	H36	H	0.354845669	0.756814036	0.451859527
C67	C	0.581076489	0.921759933	0.38602678	H37	H	0.556231891	0.588751107	0.451767042
C68	C	0.053407565	0.488013177	0.736294331	H38	H	0.850670037	0.426748329	0.246913573
C69	C	0.563125354	0.182242101	0.589314572	H39	H	0.586677938	0.451346497	0.782186716
C70	C	0.105823587	0.832578978	0.266137868	H40	H	0.80239454	0.841762684	0.943680013
C71	C	0.582589505	0.160897295	0.321308695	H41	H	0.76228794	0.049330536	0.862933348
C72	C	0.824851961	0.846247827	0.91810276	H42	H	0.88951687	0.489248163	0.563830207
C73	C	0.782696587	0.183048749	0.058254183	H43	H	0.630364743	0.871341169	0.960123431
C74	C	0.563302606	0.771791681	0.6621475	H44	H	0.776518005	0.165646357	0.127743548
C75	C	0.656880878	0.118997886	0.035878703	H45	H	0.151725959	0.092445568	0.922368892
C76	C	0.592026589	0.384163276	0.664805994	H46	H	0.060958595	0.338154442	0.692785574
C77	C	0.234235942	0.986054221	0.937198209	H47	H	0.611392046	0.995141568	0.87483748
C78	C	0.543079433	0.886789854	0.684063904	H48	H	0.582992259	0.889805332	0.268817965
C79	C	0.541684958	0.287627363	0.303515078	H49	H	0.320662831	0.183152064	0.574703782
C80	C	0.58626238	0.748169971	0.964897987	H50	H	0.140860331	0.758639452	0.298682144
C81	C	0.608355965	0.825550888	0.747699892	H51	H	0.241031204	0.233796577	0.320913354
C82	C	0.022027962	0.490459741	0.411343165	H52	H	0.332621651	0.32515453	0.027167242
C83	C	0.431651691	0.516659588	0.383847784	H53	H	0.379728084	0.043407864	0.695724313
C84	C	0.886531063	0.134102805	0.329569029	H54	H	0.715287237	0.876115374	0.334698131
C85	C	0.053716512	0.726895922	0.803807178	H55	H	0.595042233	0.151892607	0.291306182
C86	C	0.496604892	0.483639116	0.705669653	H56	H	0.78952001	0.263388537	0.401894686
C87	C	0.297046993	0.482393346	0.122737302	H57	H	0.167291238	0.790855496	0.334978226
C88	C	0.808284558	0.698599536	0.308625589	H58	H	0.529554411	0.107721147	0.913726403
C89	C	0.907641484	0.508539239	0.43924712	H59	H	0.161285322	0.71178398	0.489633555
C90	C	0.999945184	0.098303212	0.278220767	H60	H	0.185792424	0.318685365	0.515555397
C91	C	0.914413193	0.396222167	0.179601736	H61	H	0.024333202	0.699462022	0.288635108
C92	C	0.208150215	0.47135531	0.703879447	H62	H	0.711747251	0.85021161	0.758461387
C93	C	0.517162059	0.641206831	0.375366368	H63	H	0.437868721	0.421074045	0.913156843
C94	C	0.920022592	0.814487978	0.461402618	H64	H	0.203961853	0.447145065	0.178010197
C95	C	0.728849826	0.097130218	0.12976055	H65	H	0.886687879	0.642875753	0.210646149
C96	C	0.50412537	0.102652729	0.662350109	H66	H	0.369269322	0.277794093	0.435542191
C97	C	0.000567988	0.731032572	0.672814033	H67	H	0.250370755	0.977521184	0.864544852
C98	C	0.233532062	0.492681012	0.22056426	H68	H	0.260093754	0.906814359	0.563972796
C99	C	0.298178673	0.280197079	0.306607153	H69	H	0.889116121	0.199012528	0.595681932
C100	C	0.642507924	0.824193983	0.437190949	H70	H	0.350695822	0.719747426	0.569437177
C101	C	0.887050117	0.706259354	0.530940299	H71	H	0.680514115	0.653002024	0.90202549
C102	C	0.858712522	0.759635145	0.21888452	H72	H	0.33082441	0.476853798	0.12381167
C103	C	0.843999552	0.482082403	0.109384656	H73	H	0.07038993	0.855882781	0.299430278
C104	C	0.307477208	0.378528657	0.204679206	H74	H	0.153718886	0.493100642	0.437852174
C105	C	0.271663942	0.145011977	0.335124555	H75	H	0.599739806	0.293752803	0.781811516
H1	H	0.483911688	0.910177369	0.570947641	H76	H	0.989523223	0.467900636	0.706056079
H2	H	0.444566267	0.420273912	0.22001388	H77	H	0.636775436	0.706919669	0.997865204
H3	H	0.258779006	0.547717332	0.5286521	H78	H	0.556182485	0.208028233	0.703035408
H4	H	0.054241632	0.010457587	0.142488371	H79	H	0.252132109	0.56011469	0.631367469
H5	H	0.600511384	0.067454344	0.659003468	H80	H	0.861286942	0.894723784	0.958376885
H6	H	0.425546482	0.487708935	0.603906793	H81	H	0.082778337	0.965502884	0.926500357
H7	H	0.088278307	0.869052903	0.367491528	H82	H	0.247392483	0.768603547	0.701939269
H8	H	0.931185122	0.397608958	0.763652543	H83	H	0.548697522	0.519602125	0.666629179
H9	H	0.362613768	0.428203391	0.292461678	H84	H	0.533052051	0.548075294	0.471658735

H85	H	0.946853429	0.138931164	0.371909616	H160	H	0.880199844	0.683787929	0.511279013
H86	H	0.459150911	0.523694655	0.455115662	H161	H	0.447403727	0.931374627	0.874003054
H87	H	0.007778388	0.105058493	0.803043813	H162	H	0.539065356	0.819943229	0.74948614
H88	H	0.200153406	0.457096358	0.672100759	H163	H	0.967076645	0.00741393	0.879059037
H89	H	0.279547499	0.368070012	0.206473619	H164	H	0.709274193	0.349619156	0.563115564
H90	H	0.362313953	0.163549741	0.303177988	H165	H	0.641812035	0.733829968	0.218101131
H91	H	0.155819471	0.010712396	0.393659306	H166	H	0.001382103	0.915093715	0.217764331
H92	H	0.923586528	0.418330865	0.066929639	H167	H	0.792165802	0.672479488	0.322259322
H93	H	0.060835757	0.371553009	0.034995857	H168	H	0.570757959	0.53865283	0.437587326
H94	H	0.541258571	0.214202895	0.309465481	H169	H	0.595737975	0.661707291	0.840306424
H95	H	0.209706905	0.022441618	0.806781894	H170	H	0.580548468	0.705130273	0.498606476
H96	H	0.507913994	0.019126492	0.511291855	H171	H	0.889643711	0.829216074	0.459338762
H97	H	0.242930974	0.498331675	0.834678445	H172	H	0.843937924	0.495384519	0.076907757
H98	H	0.605611497	0.266750319	0.348362892	H173	H	0.729897882	0.067634221	0.312870908
H99	H	0.226779801	0.595567617	0.665741372	H174	H	0.082844474	0.735457939	0.81544313
H100	H	0.799570903	0.818528445	0.51631272	H175	H	0.714709128	0.755930906	0.389597502
H101	H	0.96983228	0.588919733	0.726916853	H176	H	0.457153301	0.884850957	0.3411005
H102	H	0.488761541	0.589345915	0.409999799	H177	H	0.35325583	0.302636721	0.183768398
H103	H	0.005806196	0.090641345	0.060060767	H178	H	0.137214565	0.360043322	0.422193104
H104	H	0.552743556	0.898697529	0.658553324	H179	H	0.761132456	0.056172773	0.713157749
H105	H	0.661473902	0.527597782	0.29474064	H180	H	0.780880004	0.650100013	0.123900503
H106	H	0.961417959	0.863327104	0.907364862	H181	H	0.094274179	0.865545389	0.264286227
H107	H	0.970136801	0.509112277	0.475741476	H182	H	0.717694633	0.968615982	0.976347068
H108	H	0.032435187	0.79770529	0.450356128	H183	H	0.533711709	0.943314016	0.094198118
H109	H	0.858208274	0.612264538	0.921822422	H184	H	0.17362273	0.890123057	0.383613561
H110	H	0.675125427	0.625623025	0.94733118	H185	H	0.021519016	0.718162032	0.652681227
H111	H	0.476555645	0.585509952	0.347400376	H186	H	0.719686338	0.773165879	0.598648364
H112	H	0.185195301	0.010554486	0.467080496	H187	H	0.215744546	0.958012179	0.942679861
H113	H	0.79466028	0.923333258	0.896223278	H188	H	0.331445373	0.724737786	0.389788598
H114	H	0.051820217	0.471501067	0.413387778	H189	H	0.711897676	0.506106869	0.291749429
H115	H	0.957305764	0.69445171	0.020504224	H190	H	0.29350631	0.559693803	0.106349358
H116	H	0.710802527	0.806283802	0.469352028	H191	H	0.48762109	0.473444364	0.674365196
H117	H	0.813428232	0.859274494	0.578260329	H192	H	0.592683194	0.352887121	0.682424173
H118	H	0.510159375	0.091921656	0.635515692	H193	H	0.59957538	0.380783227	0.365551924
H119	H	0.649276739	0.128833486	0.002862853	H194	H	0.363959307	0.993795435	0.583708256
H120	H	0.45504312	0.497323082	0.368569577	H195	H	0.28348482	0.30867091	0.319298637
H121	H	0.451149074	0.778138311	0.58812831	H196	H	0.518195702	0.526647181	0.015311495
H122	H	0.624947389	0.194662257	0.648467783	H197	H	0.713164306	0.162660876	0.353604534
H123	H	0.435306158	0.928817361	0.695994149	H198	H	0.169439983	0.628121285	0.91979239
H124	H	0.874002254	0.141305292	0.355474422	H199	H	0.500577786	0.666621712	0.224191777
H125	H	0.918156244	0.875652779	0.695464407	H200	H	0.9408117	0.439397637	0.792916804
H126	H	0.267411367	0.740543739	0.802833077	H201	H	0.842484369	0.820974898	0.476350014
H127	H	0.891413223	0.193198844	0.029928253	H202	H	0.892726725	0.646919796	0.865272163
H128	H	0.142825863	0.663341012	0.091748905	H203	H	0.532619292	0.424521804	0.668419664
H129	H	0.828271533	0.713426047	0.332437357	H204	H	0.001219468	0.491339178	0.539630965
H130	H	0.79646102	0.099821946	0.432867381	H205	H	0.010602985	0.682317546	0.726622732
H131	H	0.984717398	0.290912273	0.275637255	H206	H	0.082498332	0.738943875	0.516523302
H132	H	0.58618632	0.684450812	0.00483864	H207	H	0.938020455	0.819825496	0.433103404
H133	H	0.701099966	0.965118129	0.321506713	H208	H	0.61397012	0.49289912	0.766787164
H134	H	0.891717521	0.654756526	0.159238881	H209	H	0.061837407	0.475215105	0.765478269
H135	H	0.272819896	0.201502083	0.132480644	H210	H	0.814345915	0.413466054	0.638235382
H136	H	0.455465467	0.40904924	0.4175839	H211	H	0.117430576	0.496281625	0.186155575
H137	H	0.745591464	0.014056847	0.974856237	H212	H	0.188636185	0.822071901	0.646624892
H138	H	0.26471618	0.418864273	0.140332141	H213	H	0.055685768	0.769398691	0.742298681
H139	H	0.536112533	0.779045868	0.653966883	H214	H	0.87643483	0.105271439	0.320708573
H140	H	0.216552488	0.49328443	0.150714532	H215	H	0.181720614	0.979023948	0.796328409
H141	H	0.789032443	0.807261884	0.463785127	H216	H	0.68717655	0.473362478	0.670954093
H142	H	0.031873762	0.746366034	0.818588868	H217	H	0.440899735	0.568990771	0.432919841
H143	H	0.66159816	0.796980319	0.345326281	H218	H	0.118785846	0.139061721	0.14785856
H144	H	0.186118624	0.149734825	0.19232185	H219	H	0.556127514	0.681329275	0.70870268
H145	H	0.488969244	0.459435329	0.129804272	H220	H	0.916143008	0.266987484	0.699916183
H146	H	0.019170903	0.397586481	0.422731507	H221	H	0.998632502	0.245417183	0.777738184
H147	H	0.912280064	0.543126101	0.435919266	H222	H	0.033398434	0.179204612	0.326277796
H148	H	0.42916557	0.729467383	0.299511222	H223	H	0.291635901	0.601649765	0.4334095
H149	H	0.960415771	0.151321714	0.676951392	H224	H	0.791410649	0.907789713	0.824537607
H150	H	0.024134129	0.424059388	0.474464431	H225	H	0.252564967	0.55340539	0.777653187
H151	H	0.851134293	0.996228063	0.314291068	H226	H	0.268874387	0.661704187	0.678914752
H152	H	0.212764998	0.653488808	0.304499148	H227	H	0.145168505	0.027760633	0.92631779
H153	H	0.858420496	0.408571625	0.515295207	H228	H	0.017855055	0.653370472	0.107999508
H154	H	0.045324377	0.659224119	0.565170434	H229	H	0.183677269	0.470611662	0.724340929
H155	H	0.873209158	0.771306631	0.366881385	H230	H	0.852891373	0.620746835	0.172861843
H156	H	0.225823561	0.530756293	0.657983201	H231	H	0.074301123	0.346056922	0.817654237
H157	H	0.025757363	0.43617928	0.689779219	H232	H	0.678566503	0.843671236	0.203401837
H158	H	0.873172926	0.192328011	0.103277767	H233	H	0.877298705	0.595019237	0.232116581
H159	H	0.654358596	0.075182039	0.386967063	H234	H	0.618270167	0.802563349	0.772975093

H235	H	0.317461583	0.455761186	0.81873272	H310	H	0.782103705	0.455480286	0.357680067
H236	H	0.577854156	0.132774429	0.340233198	H311	H	0.293122594	0.59513029	0.619896551
H237	H	0.579196681	0.447182612	0.829485328	H312	H	0.387280999	0.387748743	0.384474786
H238	H	0.837868156	0.086204259	0.046634421	H313	H	0.963151106	0.451320542	0.396836796
H239	H	0.570916277	0.631722469	0.179214666	H314	H	0.449015905	0.708787385	0.495907018
H240	H	0.213966491	0.067728559	0.334119172	H315	H	0.996735571	0.074415631	0.255665441
H241	H	0.30311134	0.691021311	0.863097262	H316	H	0.014504523	0.90452589	0.373412797
H242	H	0.447662206	0.958078914	0.800460163	H317	H	0.923017516	0.518522383	0.029997814
H243	H	0.274784552	0.133498325	0.053306259	H318	H	0.865551969	0.537369732	0.970367529
H244	H	0.691156065	0.891969462	0.618867918	H319	H	0.949099958	0.284308697	0.379986161
H245	H	0.335203784	0.446427865	0.196348317	H320	H	0.279614381	0.233556922	0.362634149
H246	H	0.501794498	0.67448286	0.020955646	H321	H	0.689322697	0.12508531	0.633892193
H247	H	0.492374645	0.652239503	0.398099397	H322	H	0.15066237	0.843256852	0.409036039
H248	H	0.103789316	0.525461166	0.339819669	H323	H	0.628499755	0.605215476	0.06352693
H249	H	0.97319107	0.89530547	0.298486408	H324	H	0.167867779	0.388703032	0.740410731
H250	H	0.856258059	0.588090454	0.503644229	H325	H	0.622593018	0.796462881	0.44845222
H251	H	0.68413747	0.928297425	0.852108205	H326	H	0.796428047	0.156381633	0.051191647
H252	H	0.106493153	0.72049069	0.700544298	H327	H	0.59442188	0.530581944	0.914028239
H253	H	0.083251073	0.001551184	0.880746611	H328	H	0.604846792	0.858930116	0.760592275
H254	H	0.935693838	0.396533031	0.470167496	H329	H	0.08670606	0.145429411	0.025950519
H255	H	0.5002579	0.410883482	0.28549954	H330	H	0.02467638	0.57063835	0.304640409
H256	H	0.940155494	0.750192206	0.475423613	H331	H	0.091117209	0.332303065	0.631760312
H257	H	0.532254868	0.055795404	0.549937192	H332	H	0.335443706	0.376388431	0.887682482
H258	H	0.648051445	0.842330275	0.322674441	H333	H	0.509575025	0.004592147	0.944408671
H259	H	0.211186762	0.684708257	0.598959422	H334	H	0.109412521	0.594344419	0.79283012
H260	H	0.298192551	0.123187806	0.344600305	H335	H	0.885932601	0.278371936	0.202676818
H261	H	0.188505092	0.532829368	0.101825758	H336	H	0.563236021	0.649665499	0.236896335
H262	H	0.852034459	0.053828773	0.37807521	H337	H	0.227631488	0.583773364	0.041102118
H263	H	0.931714956	0.002017126	0.379375258	H338	H	0.814548403	0.465402085	0.113616968
H264	H	0.082568223	0.631072066	0.734267095	H339	H	0.517978715	0.242431782	0.354713449
H265	H	0.322219722	0.580892339	0.686452006	H340	H	0.142594652	0.554805863	0.689348784
H266	H	0.023306437	0.249845169	0.073965166	H341	H	0.104086322	0.95007702	0.878681233
H267	H	0.867416987	0.820211298	0.297101744	H342	H	0.737521479	0.982485463	0.781711614
H268	H	0.129997537	0.503745492	0.491928376	H343	H	0.905627374	0.496988996	0.407985493
H269	H	0.263008782	0.623150613	0.506832223	H344	H	0.679035288	0.028536999	0.201539131
H270	H	0.512812386	0.661211883	0.661331298	H345	H	0.655200982	0.064175715	0.174737695
H271	H	0.171120164	0.840349296	0.144264513	H346	H	0.248780437	0.129223895	0.318184921
H272	H	0.25283977	0.147531642	0.816359395	H347	H	0.590285008	0.301892285	0.557424925
H273	H	0.952552987	0.305924805	0.606461456	H348	H	0.135909302	0.504820307	0.322668244
H274	H	0.140451794	0.829570547	0.25672833	H349	H	0.16139988	0.015028287	0.203459795
H275	H	0.687619315	0.152347455	0.111047198	H350	H	0.988277592	0.129452949	0.264219203
H276	H	0.202220364	0.334369016	0.382688211	H351	H	0.955661731	0.812406125	0.639172663
H277	H	0.170238701	0.96414329	0.869148675	H352	H	0.944538829	0.047021045	0.382338834
H278	H	0.169065282	0.57884129	0.406077766	H353	H	0.689359692	0.260481968	0.354178824
H279	H	0.532851391	0.306235399	0.214495032	H354	H	0.721300156	0.986945244	0.331189579
H280	H	0.803877713	0.941588423	0.940702798	H355	H	0.94542148	0.406256801	0.30717587
H281	H	0.57746333	0.955552375	0.398677164	H356	H	0.623669246	0.837094498	0.65971238
H282	H	0.198148346	0.14558558	0.07744173	H357	H	0.943601062	0.202408165	0.306611617
H283	H	0.912554802	0.465185164	0.126161539	H358	H	0.415274187	0.987179358	0.601504745
H284	H	0.265349313	0.719566155	0.463334886	H359	H	0.625364296	0.586061198	0.709624736
H285	H	0.003839006	0.764683374	0.669351065	H360	H	0.201580194	0.209608026	0.190112658
H286	H	0.041417416	0.596977373	0.608649527	H361	H	0.758929205	0.083820415	0.117890641
H287	H	0.049487527	0.861896083	0.176711852	H362	H	0.393662205	0.530204503	0.071624034
H288	H	0.950635094	0.608539949	0.044961647	H363	H	0.697051249	0.288326516	0.089278749
H289	H	0.244567963	0.483618139	0.328647602	H364	H	0.641033222	0.20521521	0.324972915
H290	H	0.729956429	0.104083164	0.158675972	H365	H	0.040573532	0.515824714	0.739905509
H291	H	0.642284909	0.846111724	0.46123142	H366	H	0.472883247	0.289226925	0.855631387
H292	H	0.339030584	0.089542344	0.264963232	H367	H	0.30581251	0.540876705	0.775871976
H293	H	0.461800358	0.05531661	0.720183699	H368	H	0.56025677	0.431524219	0.03871206
H294	H	0.349368199	0.013077862	0.187569172	H369	H	0.507735018	0.356368426	0.718460015
H295	H	0.604475865	0.232510932	0.715627579	H370	H	0.665657485	0.695980477	0.732997226
H296	H	0.968100988	0.761611526	0.247037133	H371	H	0.704911945	0.076619664	0.126698459
H297	H	0.407099959	0.500504659	0.394070214	H372	H	0.528938254	0.162186742	0.693772605
H298	H	0.87659881	0.156433041	0.305257726	H373	H	0.014020973	0.229041772	0.319667035
H299	H	0.047281508	0.041828372	0.835474593	H374	H	0.550310729	0.76003289	0.63309042
H300	H	0.320992833	0.369823315	0.229600371	H375	H	0.225479082	0.437380617	0.514134028
H301	H	0.674074883	0.71791976	0.962011095	H376	H	0.918570355	0.039614558	0.417854521
H302	H	0.547230555	0.757998606	0.841185533	H377	H	0.278358476	0.598975392	0.340484241
H303	H	0.121950711	0.280062815	0.273370311	H378	H	0.843901156	0.844232684	0.730918668
H304	H	0.44222022	0.839750133	0.834417869	H379	H	0.451038738	0.56968756	0.619524001
H305	H	0.728279425	0.446701495	0.459302829	H380	H	0.582009243	0.625864856	0.884533517
H306	H	0.805395237	0.211326998	0.058452014	H381	H	0.070964673	0.810365237	0.324546981
H307	H	0.907837672	0.497203083	0.889310996	H382	H	0.877411704	0.503995932	0.457117238
H308	H	0.590624056	0.838658779	0.384604508	H383	H	0.234522867	0.403412516	0.62444021
H309	H	0.976079538	0.352296175	0.736819626	H384	H	0.836766	0.38204186	0.058022692

H385	H	0.634098603	0.283601899	0.459805213
H386	H	0.290187312	0.515724431	0.133077608
H387	H	0.854133804	0.224775199	0.982540537
H388	H	0.504008952	0.169977824	0.647906128
H389	H	0.883773379	0.444370949	0.041524971
H390	H	0.245757585	0.339634653	0.46938599
H391	H	0.287434715	0.318720348	0.452951776
H392	H	0.099713571	0.768438863	0.392211875
H393	H	0.923048587	0.429057016	0.931533687
H394	H	0.181600271	0.835545178	0.361253086
H395	H	0.797558593	0.32056599	0.689075329
H396	H	0.823593783	0.985375507	0.872831126
H397	H	0.745397147	0.001697053	0.405578916
H398	H	0.257492884	0.663361542	0.157611451
H399	H	0.590060803	0.182803943	0.740226536
H400	H	0.957679456	0.774961795	0.815326445
H401	H	0.33733048	0.227293647	0.097386374
H402	H	0.680699879	0.694063207	0.995227372
H403	H	0.018800733	0.363258996	0.66975169
H404	H	0.260773429	0.156375565	0.361216742
H405	H	0.139617399	0.672508625	0.020474922
H406	H	0.28993811	0.497767	0.717191697
H407	H	0.30133006	0.658330538	0.163405575
H408	H	0.017656404	0.423362969	0.781244291
H409	H	0.26476748	0.978453312	0.947268871
H410	H	0.632586531	0.717835464	0.344530997
H411	H	0.384127556	0.187102345	0.373707453
H412	H	0.726729906	0.099667805	0.03879398
H413	H	0.553519367	0.962452712	0.524484993
H414	H	0.627506874	0.976219309	0.995974437
H415	H	0.174925385	0.683134107	0.843991368
H416	H	0.92347644	0.368226574	0.167238374
H417	H	0.52897601	0.973070047	0.762386405
H418	H	0.862525645	0.367375196	0.104411737
H419	H	0.651615987	0.91177347	0.376049279
H420	H	0.076335695	0.688941372	0.443481369
H421	H	0.702967614	0.932433502	0.344390199
H422	H	0.431148137	0.327307705	0.329418409
H423	H	0.506827132	0.85705183	0.469748456
H424	H	0.883227764	0.779722459	0.532973944
H425	H	0.229973969	0.223132637	0.883855477
H426	H	0.934598745	0.828985911	0.486249726
H427	H	0.287939108	0.186308277	0.272915147
H428	H	0.258601986	0.602402473	0.240745207
H429	H	0.090140667	0.57140248	0.155199418
H430	H	0.727331046	0.502491966	0.353709045
H431	H	0.324745103	0.363104119	0.176959332
H432	H	0.732694187	0.83176339	0.344706049
H433	H	0.597279207	0.842652339	0.895665841
H434	H	0.812409318	0.288565167	0.777078695
H435	H	0.837290224	0.83229262	0.127194227
H436	H	0.049382631	0.866697975	0.972080168
H437	H	0.558371963	0.741770223	0.950531223
H438	H	0.63410459	0.183783798	0.375025841
H439	H	0.885545148	0.752554688	0.197937997
H440	H	0.588404586	0.824114659	0.620465927
H441	H	0.782790658	0.568534188	0.444023954
H442	H	0.427373047	0.476245784	0.966640269
H443	H	0.211496211	0.518368324	0.226433369
H444	H	0.930369175	0.269985314	0.046671262
H445	H	0.547414516	0.470370765	0.334587713
H446	H	0.936544178	0.42235721	0.179613051
H447	H	0.509613211	0.895941752	0.690880557
H448	H	0.57373829	0.922339154	0.352676362
H449	H	0.153218762	0.024400729	0.805321479
H450	H	0.524988192	0.404334318	0.925534817
H451	H	0.56779535	0.211830637	0.596637196
H452	H	0.030913796	0.09974594	0.287598191
H453	H	0.559535743	0.905046455	0.398115466
H454	H	0.290971909	0.450288839	0.224577704
H455	H	0.39219216	0.850764414	0.659753963
H456	H	0.082615063	0.4901647	0.714624527
H457	H	0.879799669	0.987532607	0.19524733
H458	H	0.442881908	0.181954225	0.391612828
H459	H	0.089788043	0.153851827	0.369744691
H460	H	0.859731306	0.381978498	0.235503002
H461	H	0.096050827	0.720603662	0.362083347
H462	H	0.640693614	0.078182779	0.314182559
H463	H	0.658578802	0.730279507	0.443148519
H464	H	0.029195643	0.596897919	0.502227458
H465	H	0.636733121	0.592687662	0.5897457
H466	H	0.548296425	0.624533775	0.926999186
H467	H	0.322441304	0.232324628	0.461151521
H468	H	0.276995139	0.782012736	0.090891049
H469	H	0.855409295	0.388085118	0.139360664
H470	H	0.472632834	0.558032633	0.09725317
H471	H	0.824725178	0.70746612	0.897230159
H472	H	0.593591854	0.590716575	0.92345375
H473	H	0.161828314	0.974231462	0.932491831
H474	H	0.086633754	0.814013983	0.248222829
H475	H	0.808551665	0.850289414	0.891869729
H476	H	0.556214056	0.540173609	0.255449473
H477	H	0.581371447	0.17418846	0.567732641
H478	H	0.929610899	0.094314133	0.97829627
H479	H	0.543379214	0.404836363	0.717821853
H480	H	0.353258516	0.579009218	0.176566655
H481	H	0.847072781	0.502921215	0.130512478
H482	H	0.610008776	0.469369986	0.683576233
H483	H	0.226942321	0.305377592	0.267012108
H484	H	0.573496422	0.272780799	0.420315349
H485	H	0.865538964	0.758598341	0.61067318
H486	H	0.055453499	0.415732441	0.930952216
H487	H	0.548512882	0.531204904	0.721861783
H488	H	0.297978667	0.928538706	0.010228776
H489	H	0.303342416	0.441687894	0.697289937
H490	H	0.160749423	0.634398573	0.970764208
H491	H	0.435668887	0.589246406	0.972851594
H492	H	0.714370606	0.394820496	0.790412243
H493	H	0.906864238	0.390415408	0.214150685
H494	H	0.334437354	0.318051005	0.828706934
H495	H	0.642351828	0.907925029	0.429035264
H496	H	0.017439047	0.503443805	0.377861388
H497	H	0.697070006	0.538749096	0.224024434
H498	H	0.529906976	0.347376357	0.535147535
H499	H	0.84529139	0.70990868	0.148890595
H500	H	0.674053903	0.252716881	0.75656277
H501	H	0.641140643	0.134265016	0.056146938
H502	H	0.880035972	0.329657439	0.271007192
H503	H	0.545006815	0.641684815	0.387001794
H504	H	0.023405289	0.516316949	0.434594444
H505	H	0.828396489	0.547029867	0.286876361
H506	H	0.714280963	0.697568672	0.938297029
H507	H	0.574073809	0.117106751	0.624782741
H508	H	0.142582052	0.629881103	0.199077593
H509	H	0.391671907	0.392794717	0.720774395
H510	H	0.335621589	0.20766628	0.384002455
H511	H	0.198983498	0.755374696	0.543988756
H512	H	0.558296206	0.220514864	0.420076595
H513	H	0.42328582	0.540467977	0.361175617
H514	H	0.716082099	0.864818379	0.386311846
H515	H	0.050846876	0.656879839	0.695633388
H516	H	0.389407918	0.828802629	0.560579894
H517	H	0.980489738	0.722859972	0.746665347
H518	H	0.941717845	0.850534799	0.831265284
H519	H	0.558611499	0.775453809	0.725963776
H520	H	0.671022577	0.814590324	0.435163454
H521	H	0.187628322	0.256937554	0.445650022
H522	H	0.862310308	0.954690191	0.876030918
H523	H	0.792185024	0.454104653	0.781251341
H524	H	0.973319219	0.173214129	0.277020627
H525	H	0.291935677	0.295401125	0.469948617
H526	H	0.253389465	0.21470402	0.625076013
H527	H	0.542646507	0.310167431	0.322398088
H528	H	0.516149526	0.284983004	0.284688707
H529	H	0.052320832	0.609412909	0.244318447
H530	H	0.183069273	0.643643742	0.518321933
H531	H	0.648813474	0.339938163	0.143165086
H532	H	0.903049588	0.832674215	0.845429905
H533	H	0.340199946	0.994954444	0.869466752
H534	H	0.865916665	0.98425469	0.914900434

H535	H	0.128409094	0.480480817	0.611507526	H610	H	0.946130771	0.066542984	0.313491608
H536	H	0.056300128	0.395218793	0.689405046	H611	H	0.421369014	0.338085153	0.996934758
H537	H	0.767255595	0.397778672	0.756870807	H612	H	0.154150771	0.667476323	0.614381545
H538	H	0.641913065	0.249603033	0.766239326	H613	H	0.29679034	0.280264623	0.276987143
H539	H	0.902430289	0.197753614	0.427647331	H614	H	0.967982439	0.434748237	0.598553365
H540	H	0.773568071	0.388990525	0.087951739	H615	H	0.049886247	0.699007386	0.811990601
H541	H	0.333507971	0.278049364	0.31422546	H616	H	0.140998088	0.044088627	0.151964272
H542	H	0.424686524	0.424695892	0.840070173	H617	H	0.899103714	0.491774393	0.355889491
H543	H	0.712112526	0.401272688	0.249635029	H618	H	0.931348655	0.847686839	0.94964546
H544	H	0.371077319	0.625453967	0.65668206	H619	H	0.426216307	0.762367016	0.897211688
H545	H	0.847419906	0.822720508	0.912305509	H620	H	0.524749239	0.395785397	0.837490764
H546	H	0.880416807	0.356937422	0.053620656	H621	H	0.665916637	0.642048582	0.045829616
H547	H	0.184562068	0.334047778	0.65539557	H622	H	0.207600514	0.545934848	0.707973202
H548	H	0.85363884	0.085395759	0.853629746	H623	H	0.997577395	0.602544088	0.535949072
H549	H	0.5112424	0.77861456	0.892026349	H624	H	0.024281922	0.350273777	0.764034706
H550	H	0.734810397	0.668669995	0.547726772	H625	H	0.598356627	0.073601308	0.562919158
H551	H	0.968518415	0.80254236	0.363793984	H626	H	0.634988096	0.445529255	0.755374276
H552	H	0.332528285	0.818337881	0.734188845	H627	H	0.898349874	0.920484876	0.887891089
H553	H	0.724999759	0.627625952	0.377120405	H628	H	0.268199022	0.040839015	0.811042475
H554	H	0.580688835	0.769246247	0.989177207	H629	H	0.628802798	0.825043398	0.722131226
H555	H	0.834435943	0.655077286	0.254223843	H630	H	0.823665587	0.735354986	0.46747709
H556	H	0.003328104	0.198909158	0.364491814	H631	H	0.841871871	0.788821807	0.205259985
H557	H	0.598729461	0.899548692	0.136208324	H632	H	0.145180876	0.732991516	0.389473077
H558	H	0.540579351	0.310672967	0.663455491	H633	H	0.133463591	0.128498077	0.857149709
H559	H	0.247823815	0.036468153	0.656002187	H634	H	0.352750971	0.707153942	0.465787768
H560	H	0.652813727	0.086366079	0.039353762	H635	H	0.519723135	0.080731399	0.685819661
H561	H	0.47164031	0.474373224	0.428988944	H636	H	0.582608473	0.38119933	0.637112521
H562	H	0.768980946	0.801250583	0.677976002	H637	H	0.560997371	0.899208254	0.710125662
H563	H	0.985001737	0.080320521	0.343438284	H638	H	0.569315299	0.290915413	0.28306215
H564	H	0.880796158	0.227799716	0.833916463	H639	H	0.101899927	0.644182173	0.683822274
H565	H	0.965521304	0.158189432	0.18821848	H640	H	0.381306019	0.817294562	0.930515512
H566	H	0.251359902	0.648816357	0.901597906	H641	H	0.232674631	0.455940566	0.718612846
H567	H	0.939563034	0.464687843	0.487614138	H642	H	0.486063157	0.474954487	0.887778301
H568	H	0.907979477	0.707722491	0.071940831	H643	H	0.036489568	0.294225424	0.979200912
H569	H	0.215993666	0.094678873	0.98232127	H644	H	0.747687296	0.844065381	0.89917573
H570	H	0.119398361	0.702770851	0.746952829	H645	H	0.807258154	0.391638627	0.8760901
H571	H	0.53559107	0.783018911	0.370841912	H646	H	0.474485063	0.506626263	0.717489978
H572	H	0.594370076	0.019010574	0.45015632	H647	H	0.96358106	0.556953195	0.666830412
H573	H	0.003037083	0.617804126	0.237847242	H648	H	0.606820807	0.443790564	0.401860149
H574	H	0.901426128	0.749292651	0.43961418	H649	H	0.976500766	0.937050984	0.992415692
H575	H	0.245615718	0.749987438	0.006042791	H650	H	0.784730926	0.720089553	0.301339055
H576	H	0.497462462	0.455903141	0.728240144	H651	H	0.961461543	0.601880216	0.95840102
H577	H	0.752280861	0.847897382	0.371187397	H652	H	0.190280383	0.118568761	0.565132327
H578	H	0.118547374	0.888636019	0.332160151	H653	H	0.904605062	0.720290729	0.31160836
H579	H	0.75741975	0.519975118	0.591752495	H654	H	0.657193512	0.23471074	0.101872895
H580	H	0.767554945	0.688211116	0.47260096	H655	H	0.34956565	0.788719086	0.616983856
H581	H	0.632784541	0.151855842	0.678055323	H656	H	0.289615082	0.586251325	0.751806048
H582	H	0.207920863	0.741388118	0.788519169	H657	H	0.262082611	0.505411225	0.221502445
H583	H	0.802267482	0.23803146	0.270349817	H658	H	0.525508662	0.816449694	0.667035493
H584	H	0.518356063	0.657053249	0.347663954	H659	H	0.21477692	0.692534045	0.39485918
H585	H	0.865874998	0.937300712	0.358091752	H660	H	0.52833262	0.012228815	0.057173344
H586	H	0.390572886	0.629240252	0.925852195	H661	H	0.305226247	0.097586798	0.469206715
H587	H	0.334261002	0.533129179	0.670450043	H662	H	0.692596068	0.734234557	0.654584091
H588	H	0.049841678	0.03657724	0.995414497	H663	H	0.866141576	0.070600865	0.624823144
H589	H	0.244796873	0.025874845	0.877810962	H664	H	0.037857235	0.413544717	0.273640552
H590	H	0.717771298	0.153949072	0.028343909	H665	H	0.373141121	0.542009813	0.49379473
H591	H	0.613459714	0.235846544	0.425884456	H666	H	0.865987609	0.79868483	0.006284763
H592	H	0.015956047	0.524117241	0.120201113	H667	H	0.294416818	0.092212957	0.217054296
H593	H	0.781450856	0.069453192	0.958538105	H668	H	0.489070708	0.211150562	0.568750695
H594	H	0.747213895	0.204612202	0.115259145	H669	H	0.603883821	0.76219269	0.943425673
H595	H	0.795603185	0.724474322	0.221360285	H670	H	0.46802075	0.104879662	0.666764773
H596	H	0.251103354	0.836664635	0.474992142	H671	H	0.776857907	0.797947259	0.824300342
H597	H	0.371300991	0.239786206	0.360071337	H672	H	0.91921749	0.70345612	0.544000793
H598	H	0.873131198	0.687051398	0.268085241	H673	H	0.9679884	0.720383894	0.660866608
H599	H	0.975602793	0.372328758	0.503079499	H674	H	0.03673685	0.306783874	0.183585941
H600	H	0.523854092	0.427777338	0.53626639	H675	H	0.136460944	0.814708168	0.648082797
H601	H	0.326300902	0.560247331	0.446165115	H676	H	0.123665064	0.223722288	0.361899669
H602	H	0.664021794	0.943085274	0.782751681	H677	H	0.183873976	0.395957632	0.377233711
H603	H	0.795360972	0.180455055	0.398782944	H678	H	0.296206225	0.545784823	0.607216163
H604	H	0.655612988	0.443868598	0.278957325	H679	H	0.75520231	0.834552943	0.105898322
H605	H	0.419770355	0.002195859	0.354432054	H680	H	0.713946883	0.893109168	0.457521116
H606	H	0.57605773	0.016012855	0.246424795	H681	H	0.28419518	0.475274776	0.091447638
H607	H	0.86705386	0.702062191	0.55696394	H682	H	0.001668419	0.017842113	0.034755387
H608	H	0.420470389	0.628776271	0.349600373	H683	H	0.852296086	0.428912206	0.177449183
H609	H	0.506731009	0.464630279	0.410907333	H684	H	0.233340285	0.045523778	0.126675746

H685	H	0.954524857	0.900893908	0.947136794	H690	H	0.810340539	0.73925808	0.515604353
H686	H	0.287920371	0.820053286	0.582261344	H691	H	0.94444754	0.903239896	0.111585809
H687	H	0.762443909	0.187954007	0.035556824	H692	H	0.564038456	0.769281358	0.695924465
H688	H	0.79731976	0.491412583	0.90109498	H693	H	0.060266934	0.436393057	0.477037345
H689	H	0.989749116	0.521138391	0.867440922	#End				

References

1) H. Nakamoto, M. Watanabe, *Chem. Commun.* **2007**, 2539-2541.

2) T. E. Faber, J. M. Ziman, *Philos. Mag.* **1965**, *11*, 153-173.

3) S. Kohara, M. Itou, K. Suzuya, Y. Inamura, Y. Sakurai, Y. Ohishi, M. Takata, *J. Phys. Condens. Matter* **2007**, *19*, 506101.

4) E. Lorch, *J. Phys. C: Solid State Phys.* **1969**, *2*, 229-237.

5) O. Gereben, P. Jóvári, L. Temleitner, L. Pusztai, *J. Optoelectron. Adv. Mater.* **2007**, *9*, 3021–3027.

6) A. L. Ankudinov, B. Ravel, J. J. Rehr, S. D. Conradson, *Phys. Rev. B* **1998**, *58*, 7565-7576.
